# Mitochondrial Mutation Leads to Cardiomyocyte Hypertrophy by Disruption of Mitochondria‐Associated ER Membrane

**DOI:** 10.1111/cpr.70002

**Published:** 2025-02-21

**Authors:** Miao Yu, Min Song, Manna Zhang, Shuangshuang Chen, Baoqiang Ni, Xuechun Li, Wei Lei, Zhenya Shen, Yong Fan, Jianyi Zhang, Shijun Hu

**Affiliations:** ^1^ Department of Cardiovascular Surgery of the First Affiliated Hospital & Institute for Cardiovascular Science, Collaborative Innovation Center of Hematology, State Key Laboratory of Radiation Medicine and Protection, Suzhou Medical College Soochow University Suzhou Jiangsu China; ^2^ Department of Endocrinology and Metabolism, Shanghai Tenth People's Hospital, School of Medicine Tongji University Shanghai Shanghai China; ^3^ Department of Obstetrics and Gynecology, Guangdong Provincial Key Laboratory of Major Obstetric Diseases, Guangdong Provincial Clinical Research Center for Obstetrics and Gynecology, Guangdong‐Hong Kong‐Macao Greater Bay Area Higher Education Joint Laboratory of Maternal‐Fetal Medicine The Third Affiliated Hospital of Guangzhou Medical University Guangzhou Guangdong China; ^4^ Department of Biomedical Engineering, School of Medicine and School of Engineering The University of Alabama at Birmingham Birmingham Alabama USA; ^5^ Department of Medicine, Division of Cardiovascular Disease, School of Medicine The University of Alabama at Birmingham Birmingham Alabama USA

**Keywords:** cardiomyocyte hypertrophy, induced pluripotent stem cell, mitochondria‐associated ER membrane, mitochondrial mutation

## Abstract

m.3243A>G is the most common pathogenic mtDNA mutation. High energy‐demanding organs, such as heart, are usually involved in mitochondria diseases. However, whether and how m.3243A>G affects cardiomyocytes remain unknown. We have established patient‐specific iPSCs carrying m.3243A>G and induced cardiac differentiation. Cardiomyocytes with high m.3243A>G burden exhibited hypertrophic phenotype. This point mutation is localised in *MT‐TL1* encoding tRNA^Leu (UUR)^. m.3243A>G altered tRNA^Leu (UUR)^ conformation and decreased its stability. mtDNA is essential for mitochondrial function. Mitochondria dysfunction occurred and tended to become round. Its interaction with ER, mitochondria‐associated ER membrane (MAM), was disrupted with decreased contact number and length. MAM is a central hub for calcium trafficking. Disrupted MAM disturbed calcium homeostasis, which may be the direct and leading cause of cardiomyocyte hypertrophy, as MAM enforcement reversed this pathological state. Considering the threshold effect of mitochondrial disease, mito‐TALENs were introduced to eliminate mutant mitochondria and release mutation load. Mutation reduction partially reversed the cellular behaviour and made it approach to that of control one. These findings reveal the pathogenesis underlying m.3243A>G from perspective of organelle interaction, rather than organelle. Beyond mitochondria quality control, its proper interaction with other organelles, such as ER, matters for mitochondria disease. This study may provide inspiration for mitochondria disease intervention.

## Graphical abstract

Cardiomyocytes with high m.3243A>G burden exhibited hypertrophic phenotype. Mitochondria dysfunction occurred and tended to become round in cardiomyocytes with high m.3243A>G burden. Mitochondria‐associated ER membrane (MAM) was disrupted in cardiomyocytes with high m.3243A>G burden. Mutation reduction and MAM enforcement partially reversed the cellular behaviour.

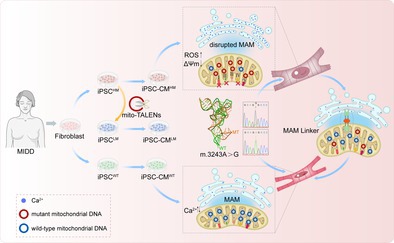

## 1 Introduction

Mitochondrial diseases comprise a diverse group of inherited disorders characterised by defects in oxidative phosphorylation [1, 2]. These defects can arise from mutations in mitochondrial DNA (mtDNA) and/or nuclear DNA. The minimum prevalence of mtDNA mutations is estimated to be 1 in 5000, while mutations in nuclear DNA related to overt mitochondrial disease are relatively low, estimated at 2.9 per 100,000 individuals [3]. Human mtDNA is a double‐stranded circular molecule, much smaller than the nuclear genome, containing 16,569 base pairs and encoding at least 37 products, including 13 structural subunits of the mitochondrial respiratory chain, 2 mitochondrial rRNAs and 22 mitochondrial tRNAs essential for the synthesis of mitochondrial proteins [4, 5]. More than half of the known pathogenic variants for mitochondrial diseases are found within tRNAs. One of the most common pathogenic point mutations is the mtDNA adenine‐to‐guanine substitution at nucleotide 3243 (m.3243A>G), located in the *MT‐TL1* gene that encodes tRNA^Leu (UUR)^. Maternally inherited diabetes and deafness (MIDD) is the most common clinical outcome in patients with m.3243A>G mutation [6]. Cardiac abnormalities are commonly reported in m.3243A>G carriers, including cardiomyopathy, arrhythmias or conduction defects. It is estimated that around 20%–40% of reported patients suffer from cardiomyopathy [7, 8]. The cardiac phenotype of m.3243A>G carriers resembles dilated cardiomyopathy, characterised by hypertrophic, concentric nonobstructive cardiomyopathy, often progressing to left ventricular dilation and systolic heart failure [7]. *MYH7*, encoding β‐myosin heavy chain, is one of the most common genes involved in hypertrophic cardiomyopathy. *MYH6*, encoding α‐myosin heavy chain, has also been reported in patients with hypertrophic cardiomyopathy, but its causal role in hypertrophic cardiomyopathy is less certain [9, 10]. There existed a transition from α‐myosin heavy chain to β‐myosin heavy chain. In patients with hypertrophic cardiomyopathy, an increase in β‐myosin heavy chain expression, that is, a higher *MYH7* to *MYH6* ratio, is commonly observed. The ratio of *MYH7* to *MYH6* was considered as cardiac hypertrophy marker. Despite significant advancements in clinical diagnosis and management, the mechanisms by which the m.3243A>G mutation leads to cardiac diseases remain to be fully elucidated.

Animal models of mitochondrial dysfunction caused by mutations in nuclear‐encoded genes have been established, while mammalian models of pathologies associated with mutations in mtDNA have been slower in development due to the difficulty of engineering the mitochondrial genome [11]. The main strategy has therefore been embryo fusion by the introduction of mitochondria carrying pathogenic mutant mtDNAs into zygotes and embryonic stem cells [12, 13, 14, 15]. The embryo fusion technique is labour‐intensive and subject to randomness during mtDNA replication. Moreover, these models usually exhibit a relatively mild phenotype compared to human patients, as clinical manifestations may involve interactions between nuclear and mitochondrial genomes. Recently, mtDNA base editors, such as DbCBE, have been developed to manipulate mtDNA precisely, though they can only catalyse C(G) to T(A) conversions [16]. Creating animal models for specific mtDNA mutations like m.3243A>G remains challenging; however, induced pluripotent stem cells (iPSCs) provide an excellent model to study m.3243A>G mutation‐induced cardiac diseases. Following reprogramming, the heteroplasmy levels in established iPSCs have demonstrated considerable stability [10]. However, studies have documented a decline in mutation levels during the initial month of culture and a rise in mutation levels over extended periods of cultivation [10, 17]. Additionally, research has highlighted clone‐specific variations in the stability of these mutation rates, underscoring the importance of routine monitoring of mutation levels in long‐term cell cultures [17, 18]. A recent study revealed that increased mitochondrial mutation heteroplasmy induces aging phenotypes in iPSCs and their differentiated progeny [19], which was much like our research scenario. Moreover, mtDNA copy number and heteroplasmy were under control of nuclear genome [20]. Therefore, mtDNA mutant heteroplasmy is decided by multiple factors. Patient‐specific iPSCs carrying mitochondrial mutations (such as m.3243A>G) can be differentiated into various cell types, including cardiomyocytes, to study tissue‐specific effects. The mutation in iPSCs reprogrammed from mutant fibroblasts kept relatively stable and could be transported to differentiated cardiomyocytes, which made it possible to conduct the following experiments. Additionally, organoids, which are 3D cell culture systems, can be created from these iPSCs to model complex tissue interactions.

As the energy powerhouse of cells, mitochondria are highly dynamic organelles that can alter their architecture to modulate interactions with other organelles. One of the best‐characterised dynamic interactions occurs between mitochondria and endoplasmic reticulum (ER), known as the mitochondria‐associated ER membrane (MAM) or mitochondria ER contact. This interaction represents approximately 10%–15% of the mitochondrial surface [21, 22]. Dozens of proteins tether MAM, maintaining a physical distance between 10 and 25 nm [23], which facilitates efficient trafficking of lipids and calcium between mitochondria and ER [24, 25, 26]. MAM has been implicated in various cellular responses, including ROS production [27, 28], ER stress [27], apoptosis [25], senescence and aging [29, 30], and cell death [31]. Dysregulation of MAM, either in the number or proteome, has been linked to neurodegenerative diseases [32, 33], diabetes [34, 35, 36] and cardiovascular diseases [37, 38, 39, 40]. The development of electron microscopy and machine learning has enabled quantitative analysis of MAM, bridging MAM dynamism to physiological and pathological processes [41].

In this study, we have established iPSCs from the m.3243A>G carriers diagnosed with MIDD and differentiated them into cardiomyocytes (iPSC‐derived cardiomyocytes [iPSC‐CMs]) to characterise phenotypes and investigate involved mechanisms. This patient was diagnosed with MIDD and predisposed to heart disease. We found that iPSC‐CMs with a high m.3243A>G mutation rate exhibited hypertrophy with disorganised sarcomere and a subsequent senescence phenotype. Alongside oxidation phosphorylation defects, mitochondria became rounded, and their interaction with ER was weakened, as evidenced by decreased contact number and length between these organelles, indicating disruption of MAM. Consequently, calcium trafficking was impaired, and calcium homeostasis was imbalanced. Both mutation reduction and MAM reinforcement could reverse the phenotype. Therefore, we concluded that m.3243A>G mutation is associated with cardiomyocyte hypertrophy via disruption of MAM. Beyond mitochondria itself, their interaction with ER may represent a novel target for treating mitochondrial cardiomyopathy.

## 2 Materials and Methods

### 2.1 hiPSC Generation

In this study, we sampled a maternally inherited family with clinically diagnosed MIDD of the mother and daughter carrying the m.3243A>G mutation. After obtaining approval from the ethics committee of Soochow University and securing informed consent from the patient, a 2 mm diameter skin tissue sample was collected. Skin fibroblasts were isolated by digesting the tissue with Collagenase I (1 mg/mL) at 37°C for 4 h and then cultured in a fibroblast culture medium. When the fibroblast density reached approximately 80%, the cells were transduced with lentiviral particles containing human POU5F1, SOX2, KLF4 and c‐MYC, as previously described with slight modifications [42]. Seven days post‐transduction, the infected cells were plated onto dishes coated with MEF cells. Visible cell clones were manually picked under a microscope after approximately 25 days, transferred to new Petri dishes, and passaged for subsequent experiments. Achieved hiPSCs complied with the standards of stem cells and human‐iPSCs published previously [43, 44].

### 2.2 Teratoma Formation

Teratoma assays are considered the gold standard for demonstrating the differentiation potential of pluripotent stem cells. About 2 × 10^5^ of the three iPSC lines mixed with Matrigel were injected subcutaneously into immunodeficient mice. After 1 month, differentiated tumours were formed. All mice were euthanised by cervical dislocation under 3% isoflurane anaesthesia. Teratomas were excised and fixed with 4% PFA (Sigma‐Aldrich, USA). H&E staining (Beyotime, China) was performed after paraffin sectioning to see whether it comprising all the three germ layers (endoderm, mesoderm and ectoderm), which can indicate the pluripotency of iPSC.

### 2.3 Karyotype Analysis

Karyotyping of hiPSCs was performed by the Chromosome Laboratory in the First Affiliated Hospital of Soochow University. In brief, the cells in their exponential growth phase were harvested. After treatment with prewarmed hypotonic solution (KCl), the cells were fixed with acetic acid/methanol solution at a ratio of 1:3. The cells were then dropped onto slides for chromosome analysis using the trypsin‐Giemsa banding technique. The representative images were recorded.

### 2.4 m.3243A>G Mutation Rate Analysis

The mutation rate was examined by PCR‐PFLP. Whole‐genome DNA was extracted, and a fragment with a length of 550 bp flanking nucleotide 3243 of mitochondria genome was amplified using forward primer (5′‐CCTCCCTGTACGAAAGGACA‐3′) and reverse primer (5′‐CACCCTGATCAGAGGATTGAG‐3′). After purification, the DNA product was digested with endonuclease ApaI (New England Biolabs, USA) and separated by 1% agarose gel electrophoresis. PCR products containing the m.3243A>G mutation were digested into two fragments with different sizes (420 and 130 bp), while those wild‐type (WT) ones could not be cut. The signal intensity of the DNA bands was analysed using ImageJ software.

### 2.5 hiPSC Maintenance and Cardiomyocyte Differentiation

hiPSCs were routinely cultured using PSCeasy E8 medium (Cellapy, China). When the cell confluence reached approximately 70%, the cells were passaged using 0.5 mM EDTA (Sigma‐Aldrich, USA) and seeded into a Petri dish coated with Matrigel (Corning, USA). During this process, 2 mM thiazovivin (Selleck Chemicals, USA), a rho‐related protein kinase inhibitor, was added to inhibit cell apoptosis and promote cell attachment. When the cell density reached approximately 80%, cardiomyocyte differentiation was initiated in the CDM3 medium as previously described [45]. The cells were treated with 4 μM CHIR99021 (Sigma‐Aldrich, USA) for two consecutive days, followed by 2 μM Wnt‐C59 (Sigma‐Aldrich, USA) for 1 day. The medium was then refreshed daily. Beating cardiomyocytes could be observed around Day 8. On Day 12, cardiomyocytes were purified using glucose‐free RPMI 1640 medium (Thermo Fisher, USA) supplemented with 5 mM sodium dl‐lactate (Sigma‐Aldrich, USA) for three consecutive days. For further analysis, cardiomyocytes were then split using 0.25% trypsin (Sigma‐Aldrich, USA) containing 0.1 mM EDTA (Sigma‐Aldrich, USA) and seeded on dishes coated with 0.1% gelatin (Sigma‐Aldrich, USA) in CDM3 medium. Cardiomyocytes at Day 30 of induction were used for experiments. hiPSC‐CMs meet the standard of human cardiomyocytes published previously [46].

### 2.6 Flow Cytometry

Differentiated cells were dissociated into single cells with 0.25% trypsin–EDTA and fixed in 1% PFA for 10 min. Specific primary antibodies were used for flow cytometry analysis. Data were acquired and analysed using Guava easyCyte 8HT (Millipore, Germany). Antibodies used are listed in Table S1.

### 2.7 Cardiac Organoid Generation

2 × 10^5^ iPSCs were seeded into low attachment dishes to form EB spheroids. After that, EB spheroids were sequentially treated with CHIR99021 for 2 days, bFGF (Novoprotein, USA), BMP4 (Peprotech, USA) and VEGF (Novoprotein, USA) for 6 days, and then bFGF and VEGF for 2 days to form vascular organoids. The next day, vascular organoids were transplanted into a new low attachment dish and co‐cultured with 1 × 10^5^ differentiated cardiomyocytes to obtain cardiac organoids.

### 2.8 Masson Trichrome Staining

A Masson's Trichrome stain kit (Solarbio, China) was adopted to detect the fibrosis of cardiac organoids, with ponceau and aniline blue staining muscle fibre and collagen fibre, respectively. The paraffin sections were photographed and analysed.

### 2.9 Immunofluorescence Staining

Samples were fixed in 4% PFA to preserve cell structure and antigenicity, incubated with specific primary antibodies overnight at 4°C, washed two times with PBST, incubated with the corresponding secondary antibodies for 1 h at room temperature, and nuclei were stained with Hoechst 33342 (Thermo Fisher, USA). Labelled cells were examined and imaged with a confocal microscope (Zeiss, Germany). Antibodies used for immunofluorescence are listed in Table S1.

### 2.10 β‐Gal Senescence Staining

Senescence was detected using a β‐galactosidase staining kit (Beyotime, China). Cells were fixed and then incubated with the staining working solution at 37°C overnight in a CO_2_‐free incubator and observed under the microscope on the next day.

### 2.11 Real‐Time PCR

Total RNA was extracted using TRIzol reagent (Thermo Fisher, USA) and reverse transcribed into cDNA using the PrimeScript RT kit (Takara, Japan). SYBR Green was used in the ABI StepOnePlus Real‐Time PCR System (Thermo Fisher, USA). Data were analysed using the 2^−ΔΔCT^ method. Primers used for real‐time PCR are listed in Table S2.

### 2.12 Western Blot

Proteins were extracted using RIPA lysis buffer (Beyotime, China), quantified by BCA assay (Beyotime, China), separated by SDS‐PAGE, and transferred onto PVDF membranes (EMD Millipore, Germany). Membranes were blocked by 5% skimmed milk for 1 h at room temperature, incubated with specific primary antibodies overnight at 4°C, followed by corresponding secondary antibodies for 1 h at room temperature, and developed with ECL substrate (Cell Signaling Technology, USA). Images were captured with a ChemiDoc XRS system (Bio‐Rad, USA). Antibodies used for western blot are listed in Table S1.

### 2.13 Measurement of Intercellular ROS by DCFH‐Based Flow Cytometry

The fluorescent probe DCFH‐DA (Beyotime, China) was used to measure intercellular ROS levels. After entering cells, non‐fluorescent DCFH‐DA hydrolysed to DCFH and then oxidised into fluorescent DCF by intercellular ROS. Briefly, the cells were incubated with a prewarmed medium containing 0.1 mM DCFH‐DA at 37°C for 20 min, followed by fluorescence intensity measurement using flow cytometry (Millipore, Germany).

### 2.14 Simulation Analysis of tRNA Molecular Dynamics

The mtDNA sequence containing the mutation site underline displayed (GUUAAGAUGGCAGAGAGCCCGGGUAAUCGCAUAAAACUUAAAACUUUACAGUCAGAGAGGUUUCAAU CCUCUUCUUUAACA) was used to generate the topology and the dot‐bracket secondary structure of tRNAs by RNAcentral (https://rnacentral.org/). The 3D structure of the target tRNA sequences was constructed for subsequent molecular dynamics simulations using the GROMACS software package with the RNA OL3 force field parameters. The tRNA was placed in a cubic simulation box and solvated with the TIP3P explicit water model, setting periodic boundary conditions with the box boundary at a minimum distance of 1 nm from the nearest atom of the tRNA. The molecular dynamics simulation workflow consisted of four steps: energy minimisation, heating, equilibration and production dynamics simulation. First, constraining the heavy atoms of the tRNA, energy minimisation was performed for 10,000 steps, including 5000 steps of steepest descent method and 5000 steps of conjugate gradient method; subsequently, the constraints were released and the entire system underwent 10,000 steps of energy optimisation. After energy minimisation, the system was gradually heated to 300 K over 50 ps; following heating, the system was equilibrated for 50 ps under the NPT ensemble. Finally, the system underwent a 100 ns molecular dynamics simulation under the NPT ensemble with a time step of 2 fs. Trajectory data were saved every 20 ps for subsequent data analysis.

### 2.15 Metabolic Assay

A Seahorse XF24 Analyser (Agilent, USA) was used to measure oxygen consumption rate (OCR) and extracellular acidification rate (ECAR). OCR was measured by sequentially adding 2 μM oligomycin, 1 μM FCCP and 0.5 μM rotenone/antimycotic A. ECAR was measured by sequential treatment of 10 mM glucose, 2 μM oligomycin and 50 mM 2‐DG. Both were normalised to protein concentration.

### 2.16 Measurement of Mitochondrial Membrane Potential

A mitochondrial membrane potential assay kit with JC‐1 (Beyotime, China) was used to detect the changes in mitochondrial membrane potential. When the mitochondrial membrane potential is high, JC‐1 exists in the form of aggregates and emits red fluorescence. When the mitochondrial membrane potential is low, JC‐1 exists in the form of monomers and emits green fluorescence. Mitochondrial membrane potential was measured by the ratio of the red fluorescence to the green fluorescence. As a decoupler, CCCP can eliminate the mitochondrial membrane potential and be used as a positive control. Briefly, the cells were treated with JC‐1 staining solution at 37°C for 20 min and then washed two times with JC‐1 buffer. Images were taken, and fluorescence intensity was statistically analysed.

### 2.17 Measurement of Mitochondrial ROS

The mitochondrial superoxide indicator MitoSOX (Invitrogen, USA) was used to detect superoxide production in mitochondria. The cells were treated with 1 μM dye diluted in HBSS buffer at 37°C for 30 min, followed by fluorescence intensity analysis.

### 2.18 Tracking of Mitochondria and ER

Mito‐Tracker Red (Invitrogen, USA) and ER‐Tracker Green (Invitrogen, USA) were used to indicate mitochondria and ER, respectively. After nuclear staining with Hoechst 33342, the cells were visualised under an LSM 880 confocal microscope (Zeiss, Germany).

### 2.19 Deep Analysis of the Interaction Between ER and Mitochondria

The quantitative analysis of MAM by electron microscopy and deep learning DeepContact was performed as previously described [41]. Sapphire disks (Wulundes, China) were placed on the bottom of the cell culture plate and coated with Matrigel. iPSC‐CMs were seeded on it. After 3–4 days of culture, slides were fixed in 2.5% (vol/vol) glutaraldehyde in phosphate buffer (0.1 M, pH 7.4). After fixation, cell samples were transferred to the Center for Biological Imaging, Institute of Biophysics, Chinese Academy of Sciences (Beijing, China) for the following sample preparation by the reduced osmium‐thiocarbohydrazide‐osmium method to highlight the organelle outline by preferential staining of lipid bilayers and subsequently benefit the recognition of organellar boundaries. Samples cut into ultrathin sections (70‐nm‐thick) underwent scanning electron microscopy. Images at 5‐nm resolution were acquired in the main cytoplasmic area near the nuclei for the DeepContact Analysis. DeepContact analysis can be divided into five stages: preprocessing, mitochondria segmentation, ER segmentation, visualisation and MAM quantification.

### 2.20 Calcium Transient

The fluorescent calcium indicator Fluo‐4 AM (Thermo Fisher, USA) was used to label intercellular free calcium. The cells were treated with Fluo‐4 AM for 30 min at 37°C and washed two times with DPBS for 5 min each to remove the non‐specifically bound dye. Intracellular calcium ion fluxes were captured at a sampling rate of 0.5 ms/line. Beat frequency was recorded. Time to peak and decay time were statistically analysed.

### 2.21 MAM Linker

The synthetic ER–mitochondria linker plasmid was constructed as previously described [47]. Briefly, one at the N‐terminal end encoding AKAP1 is linked to the mitochondria, one at the C‐terminal end encoding UBC6 is linked to the ER and the middle segment is mRFP, which can maintain distance between the mitochondria and ER.

### 2.22 Statistical Analysis

Comparisons between the two groups were analysed using Student's *t*‐test. Multiple comparison correction analysis was performed using ANOVA with the Bonferroni post hoc test. Statistical significance was indicated by *p* < 0.05. Data are presented as the mean ± SEM.

## 3 Results

### 3.1 The m.3243A>G Mutation Induced Cardiomyocyte Hypertrophy

We have sampled a mother and daughter who have been diagnosed with diabetes before. The existence of hearing disturbance and short stature prompted us to make further diagnoses. Sanger sequencing revealed an adenine‐to‐guanine mutation at position 3243 of mtDNA (m.3243A>G) (Figure S1). This confirmed a definite diagnosis of MIDD. To explore the disease mechanism, we have established their specific iPSCs by fibroblast reprogramming. In addition to the abovementioned clinical symptoms, anomaly indices with elevated NT‐proBNP (278.20 pg/mL)/hs‐TnT (0.02 ng/mL) and T‐wave flatness were exhibited. This prognostic prompted an investigation into the effects of the m.3243A>G mutation on cardiac and its underlying mechanism using iPSC‐CMs.

The patient's skin fibroblasts were isolated and reprogrammed into iPSCs. Due to the heteroplasmy of mtDNA, we obtained two iPSC lines: iPSC^HM^ with a high m.3243A>G mutation rate (~80%) and iPSC^WT^ without the mutation (Figures S2 and S3A,B). Mitochondrial diseases occur only when the heteroplasmy level exceeds a certain threshold, typically > 80% [48]. Previously, we have engineered mitochondrial‐targeted transcription activator‐like effector nucleases (mito‐TALENs) and successfully eliminated the m.3243A>G mutation in iPSC from patients diagnosed with mitochondrial encephalomyopathy and stroke‐like episodes [49]. In this study, mito‐TALENs were also introduced to eliminate mutant mtDNA targeted m.3243A>G to establish another line iPSC^LM^ with reduced mutation burden (~30%) (Figures S2 and S3B). The mutation rate was estimated using PCR‐RFLP. Fragments spanning tRNA^Leu (UUR)^ were amplified by PCR and subsequently digested with Apa I, which cleaves at the site of the m.3243A>G (Figure S3C,D). The mutation rate remained stable over continuous passages (Figure S3E).

After confirming the pluripotency of the iPSC lines (Figure S4A–E), we differentiated them into cardiomyocytes (Figure S5A), named iPSC‐CM^WT^, iPSC‐CM^HM^ and iPSC‐CM^LM^, respectively. Flow cytometry results showed that m.3243A>G mutation does not affect the differentiation from iPSC into cardiomyocytes (Figure S5B,C), and the mutation rate was maintained during differentiation (Figure S5D). However, cardiomyocytes with the m.3243A>G mutation showed reduced and weakened spontaneous beating (Movies [Link], [Link]).

**FIGURE 1:**
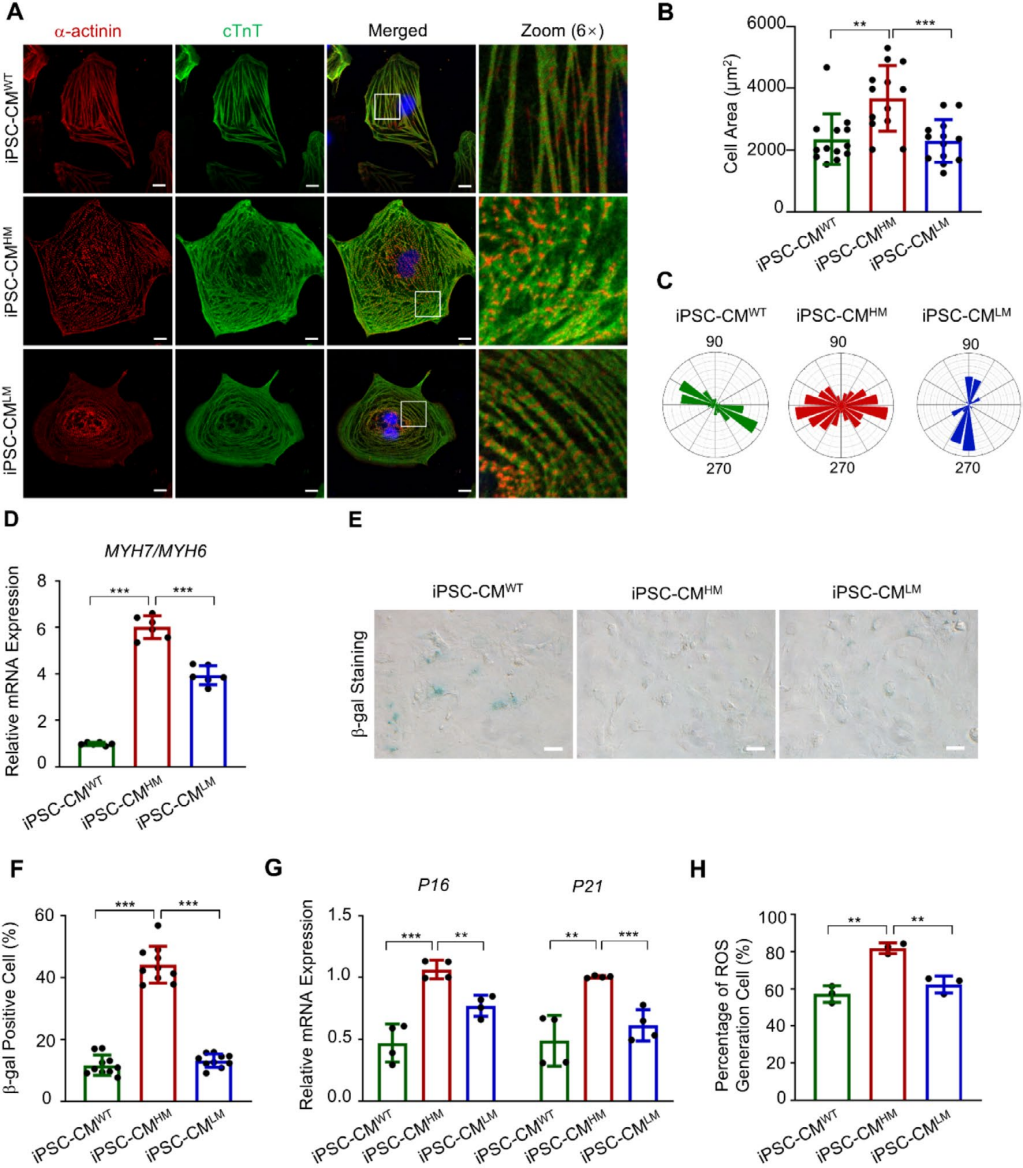
m.3243A>G mutation induced cardiomyocyte hypertrophy. (A) Immunofluorescence staining of a‐actinin (red) and cTnT (green) to display sarcomeres with Hoechst 33342 (blue) indicating nuclei at Day 30 post‐differentiation. Magnified views of the boxed areas in the merged micrographs showed the detailed α‐actinin and cTnT staining patterns. Scale bar, 10 μm. (B) Statistical analysis of cell area showed that iPSC‐CM^HM^ exhibited a significantly larger cell area compared to iPSC‐CM^WT^, while the cell area in iPSC‐CM^LM^ is similar to that in iPSC‐CM^WT^. (C) Representative polar histogram of the sarcomeric orientation showed less alignment in iPSC‐CM^HM^ compared to iPSC‐CM^WT^ and iPSC‐CM^LM^. (D) mRNA expression ratio of *MYH7* to *MYH6*. (E) β‐gal staining illustrated cell senescence. Scale bar, 50 μm. (F) Statistical analysis of β‐gal positive cells indicated that iPSC‐CM^HM^ exhibited a significantly higher rate compared to iPSC‐CM^WT^ and iPSC‐CM^LM^. (G) Real‐time PCR detection of mRNA expression of *P16* and *P21*. (H) Statistical analysis of intercellular ROS levels detected by flow cytometry. Data are presented as mean ± SEM; one‐way ANOVA; **p < 0.01, ***p < 0.001.

The stable mutation rate and comparable differentiation rate enabled us to assess the influence of the m.3243A>G mutation on cardiomyocyte structure and function. As shown in Figure 1A,B, cardiomyocytes with a high m.3243A>G mutation rate exhibited hypertrophy, indicated by increased surface area and disorganised sarcomeres. Polar histogram statistics showed disordered sarcomere orientation in iPSC‐CM^HM^ compared to iPSC‐CM^WT^, which was improved in iPSC‐CM^LM^ (Figure 1C). The ratio of *MYH7* to *MYH6* was also upregulated in mutant cells (Figures 1D and S6A), which was considered as cardiac hypertrophy marker [50, 51]. Given that cardiac hypertrophy is a detrimental feature of cardiac ageing [52], we investigated whether m.3243A>G mutation accelerated the ageing process of cardiomyocytes. As shown in Figure 1E,F, iPSC‐CM^HM^ exhibited an apparent cellular senescence trend, the primary ageing process at the celluar level [53, 54], indicated by a higher positive rate of β‐gal. Concomitantly, expression levels of senescence‐associated *P16* and *P21* were higher in iPSC‐CM^HM^ (Figure 1G). Additionally, iPSC‐CM^HM^ produced more reactive oxygen species (ROS) (Figures 1H and S6B). Cardiac organoids from iPSC^HM^ also exhibited larger size compared to those from iPSC^WT^ (Figure S6C). This phenotype, from the perspective of three dimensions, proved the aforementioned hypertrophic trend. Fibrosis is universally observed in multiple ageing‐related diseases. Compared to WT ones, cardiac organoids from iPSC^HM^ were vulnerable to fibrosis, as indicated by Masson's Trichrome staining (Figure S6C,D). After the reduced mutation load, the phenotype of cardiomyocytes became more similar to that of control ones. Taken together, the m.3243A>G mutation induced cardiomyocyte hypertrophy and accelerated the ageing process, potentially contributing to cardiac dysfunction in carriers of the mutation. However, the underlying mechanism requires further investigation.

### 3.2 The m.3243A>G Mutation Destabilised tRNA^Leu^ ^(UUR)^ and Malfunctioned Mitochondria

**FIGURE 2:**
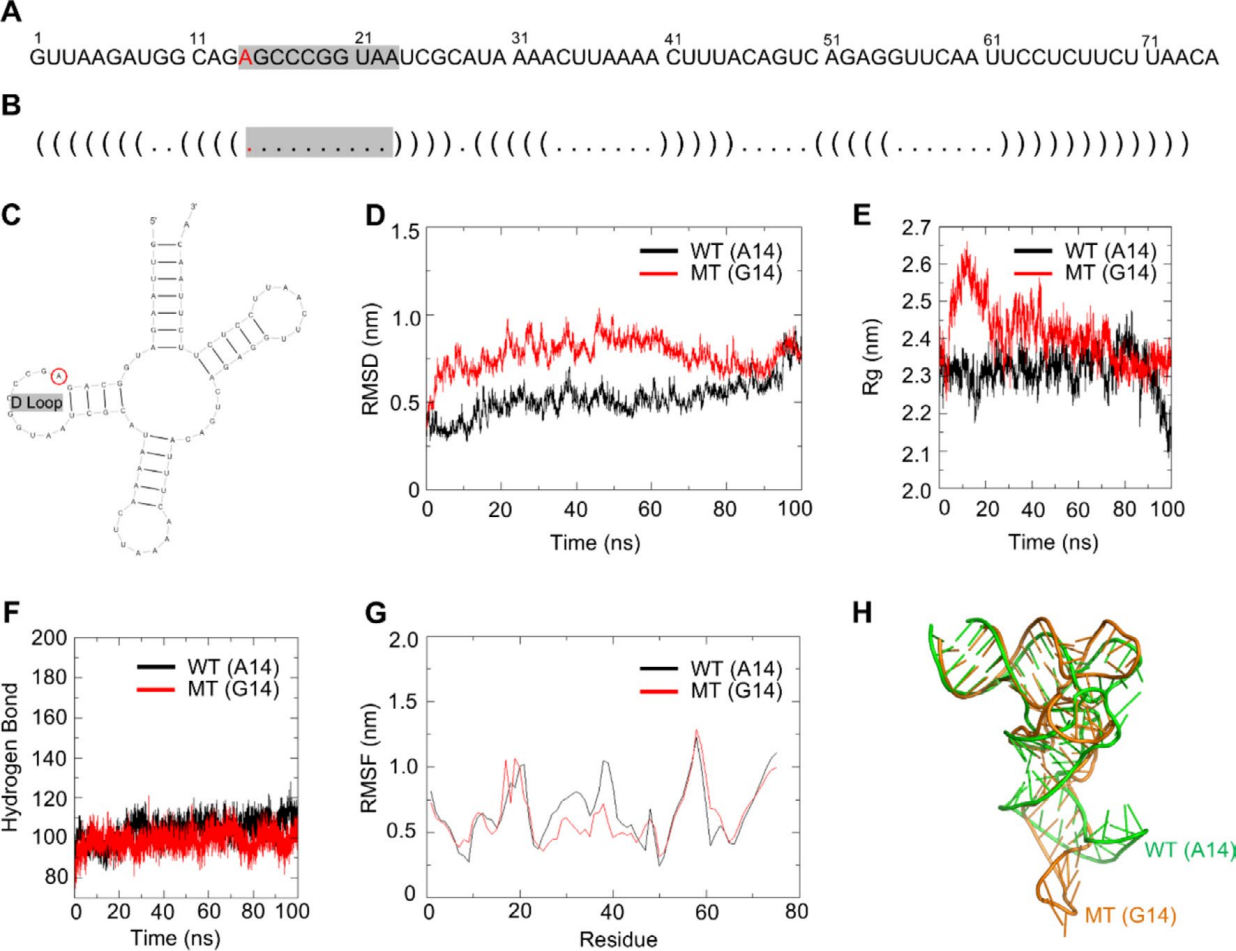
m.3243A>G mutation altered tRNA^Leu (UUR)^ transformation and stability. (A) The nucleotide sequence of tRNA^Leu (UUR)^ with mutated A at position 14 highlighted in red. (B) The secondary structure of tRNA^Leu (UUR)^ displayed in dot‐bracket notation with mutation site marked in red. (C) The secondary structure of tRNA^Leu (UUR)^ displayed in a cloverleaf model, with the mutated A (circled in red) in the D‐Loop. (D) Time evolution of the root mean square deviation (RMSD) values for the wild‐type (WT) (black curve) and mutant (MT) (red curve) of tRNA^Leu (UUR)^. (E) Time evolution of the radius of gyration (Rg) values for the WT (black curve) and MT (red curve) of tRNA^Leu (UUR)^. (F) The number of hydrogen bonds for the WT (black curve) and MT (red curve) of tRNA^Leu (UUR)^. (G) The root mean square fluctuation (RMSF) curves for the WT (black lines) and MT (red lines) of tRNA^Leu (UUR)^. (H) Schematic model of tertiary structure for the WT (green) and MT (brown) tRNA^Leu (UUR)^.

We aimed to elucidate the mechanism underlying the effects of m.3243A>G point mutation. This mutation occurs in the mtDNA at position 3243, within the *MT‐TL1* gene that encoded tRNA^Leu (UUR)^. Specifically, the mutation changes the nucleotide at position 14 (A14) to guanine (G14) within the D‐loop of tRNA^Leu (UUR)^ (Figure 2A–C). Molecular dynamics simulations were conducted to assess the impact of m.3243A>G mutation on the tertiary structure of tRNA^Leu (UUR)^. The mutation tRNA (G14) exhibited increased structural instability, as evidenced by the greater fluctuation in the root mean square deviation (RMSD) curve, a larger radius of gyration (Rg), a reduced number of hydrogen bonds, and higher root mean square fluctuation (RMSF) compared to WT (A14) tRNA (Figure 2D–G). These alterations led to a more relaxed tertiary structure (Figure 2H).

**FIGURE 3:**
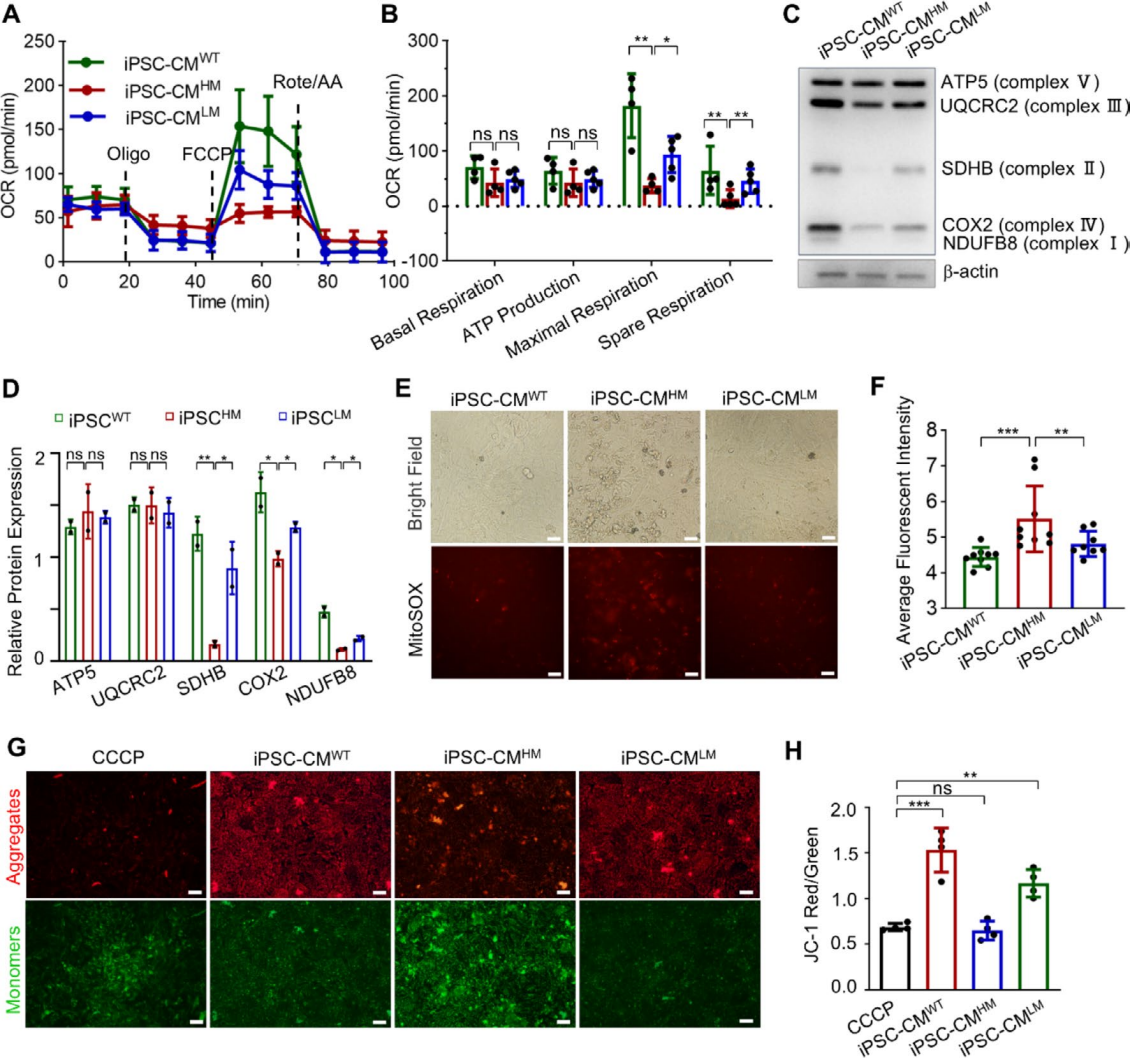
m.3243A>G mutation impaired mitochondrial function. (A) Seahorse XF assay was used to evaluate OCR with sequential treatment of oligomycin (Oligo), carbonyl cyanide 4‐(trifluoromethoxy) phenylhydrazone (FCCP), and rotenone and antimycin A (Rote/AA). (B) Statistical analysis of basal respiration, ATP production, maximal respiration, and spare respiration. (C) Western blot of five OXPHOS subunits: NDUFB8 (Complex I), SDHB (Complex II), UQCRC2 (Complex III), COX2 (Complex IV) and ATP5 (Complex V). (D) Statistical analysis of the relative protein expression of five OXPHOS subunits in (C). (E) MitoSox staining indicated mitochondrial ROS accumulation. Scale bar, 100 mm. (F) Statistical analysis of mitochondrial ROS accumulation. (G) Immunostaining of JC‐1 Aggregates (Red) and JC‐1 Monomers (Green) indicated mitochondrial membrane potential, with CCCP‐treated cardiomyocyte as control. Scale bar, 100 mm. (H) Statistical analysis of the ratio of JC‐1 Aggregates (Red) to JC‐1 Monomer (Green). Data are presented as mean ± SEM; one‐way ANOVA; *p < 0.05, **p < 0.01, ***p < 0.001, ns: not significant.

Given that tRNA mutations are the primary causes of mitochondrial dysfunction [55], we investigated the influence of m.3243A>G mutation on mitochondrial function in cardiomyocytes. Mitochondrial oxidative phosphorylation (OXPHOS) activity was measured by an XF‐24 Extracellular Flux Analyser, indicated by the OCR. Mutant cells showed a significant decrease in both maximal and spare respiration compared to control cells. Partial restoration of OXPHOS activity was observed after reducing the mutation burden. Additionally, both basal and ATP‐linked OCRs in iPSC‐CM^HM^ exhibited a downward trend (Figure 3A,B). OXPHOS involves five complexes composed of subunits encoded by both mtDNA and nuclear DNA. To determine whether the mutation affected the respiratory chain, we analysed the levels of key subunits from each complex using an optimised antibody cocktail targeting Complex I subunit NDUFB8, Complex II subunit SDHB, Complex III subunit UQCRC2, Complex IV subunit COX2 and Complex V subunit ATP5. The iPSC‐CM^HM^ showed severe deficiencies in Complexes I, II and IV (Figure 3C,D). Notably, COX2, encoded by mtDNA, was particularly affected, suggesting a direct impact of the m.3243A>G mutation. The nuclear‐encoded subunits NDUFB8 and SDHB were also impacted, indicating possible interactions between mtDNA mutations and the nuclear genome, warranting further investigation.

To compensate for energy deficits, iPSC‐CMs with high m.3243A>G mutation rates increased in glycolysis, as indicated by higher ECAR (Figure S7A,B). This shift in mitochondrial respiration to glycolysis is less efficient for energy production and correlated with a decreased beat rate for iPSC‐CM^HM^ (Movie S2). Moreover, there was an overproduction of ROS (Figure 3E,F) and a reduction in membrane potential (Figure 3G,H), indicative of mitochondrial dysfunction. Elevated glycolysis also led to increased lactic acid production and upregulated lactylation (Figure S7C), which may influence nuclear gene expression. Under these conditions, mitochondria appeared more rounded rather than the typical rod shape (Figure S7D), suggesting possible impacts on organelle interactions such as with the ER. We have also detected mitochondria dynamics and mitophagy. The decreased expression of MFN1 and MFN2 and the unaffected p‐DRP1/DRP1 revealed the imbalance in mitochondria fusion/fission in cardiomyocyte‐carrying mutation, which may reshape mitochondria and influence its interaction with ER (Figure S7E).

### 3.3 The m.3243A>G Mutation Disrupted MAM and Calcium Homeostasis

**FIGURE 4:**
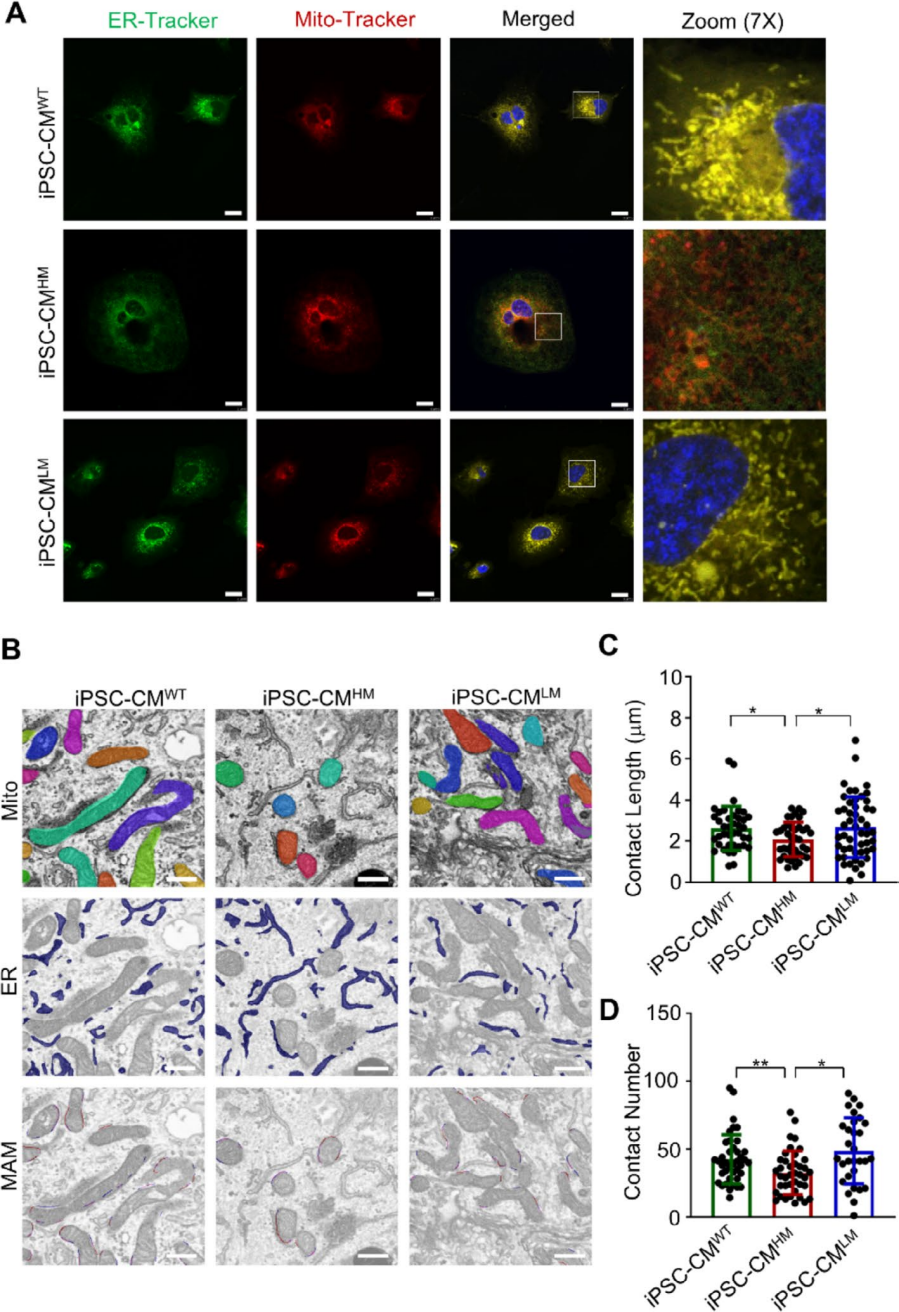
m.3243A>G mutation disrupted MAM. (A) ER, mitochondria, and nucleus are labelled in green, red, and blue, respectively. The overlapping of green and red signals merged in yellow indicated MAM. Scale bar, 4 mm. (B) Machine learning DeepContact was used to identify mitochondria and ER and characterise MAM. Scale bar, 1 mm. (C) Quantification of contact length between mitochondria and ER. (D) Quantification of contact number between mitochondria and ER. Data are presented as mean ± SEM; one‐way ANOVA; *p < 0.05, **p < 0.01.

The m.3243A>G mutation has notable impacts on the structure and function of mitochondria, prompting an investigation into its effect on MAMs. To explore this, we employed fluorescence immunostaining to visualise ER (in green) and mitochondria (Mito, in red). The overlap of these signals, indicating MAM, appears yellow. As shown in Figure 4A, the interaction between ER and mitochondria was significantly reduced in iPSC‐CM^HM^ cells compared to that in iPSC‐CM^WT^ cells. After reducing the mutation burden, the yellow fluorescence signalling in iPSC‐CM^LM^ showed partial recovery.

For a qualitative assessment, we utilised machine learning. A scanning electron microscope was employed to capture ultra microstructures of cardiomyocytes derived from iPSCs with varying levels of m.3243A>G mutation. We then used DeepContact, a cutting‐edge deep‐learning protocol, to identify and segment organelles [41]. The contact between mitochondria and ER was visualised and quantified, defining MAM as regions where the distance between mitochondria and ER ranged from 10 to 25 nm. As illustrated in Figure 4B, cardiomyocytes with a high m.3243A>G mutation rate exhibited weakened mitochondria–ER interactions, as evidenced by reduced contact length and number (Figure 4C,D). This interaction was partially restored upon mutation load reduction, with parameters resembling WT cells. Additionally, we observed fewer mitochondria with shorter lengths, suggesting an imbalance in mitochondrial fusion and fission (Figure S8A–C).

**FIGURE 5:**
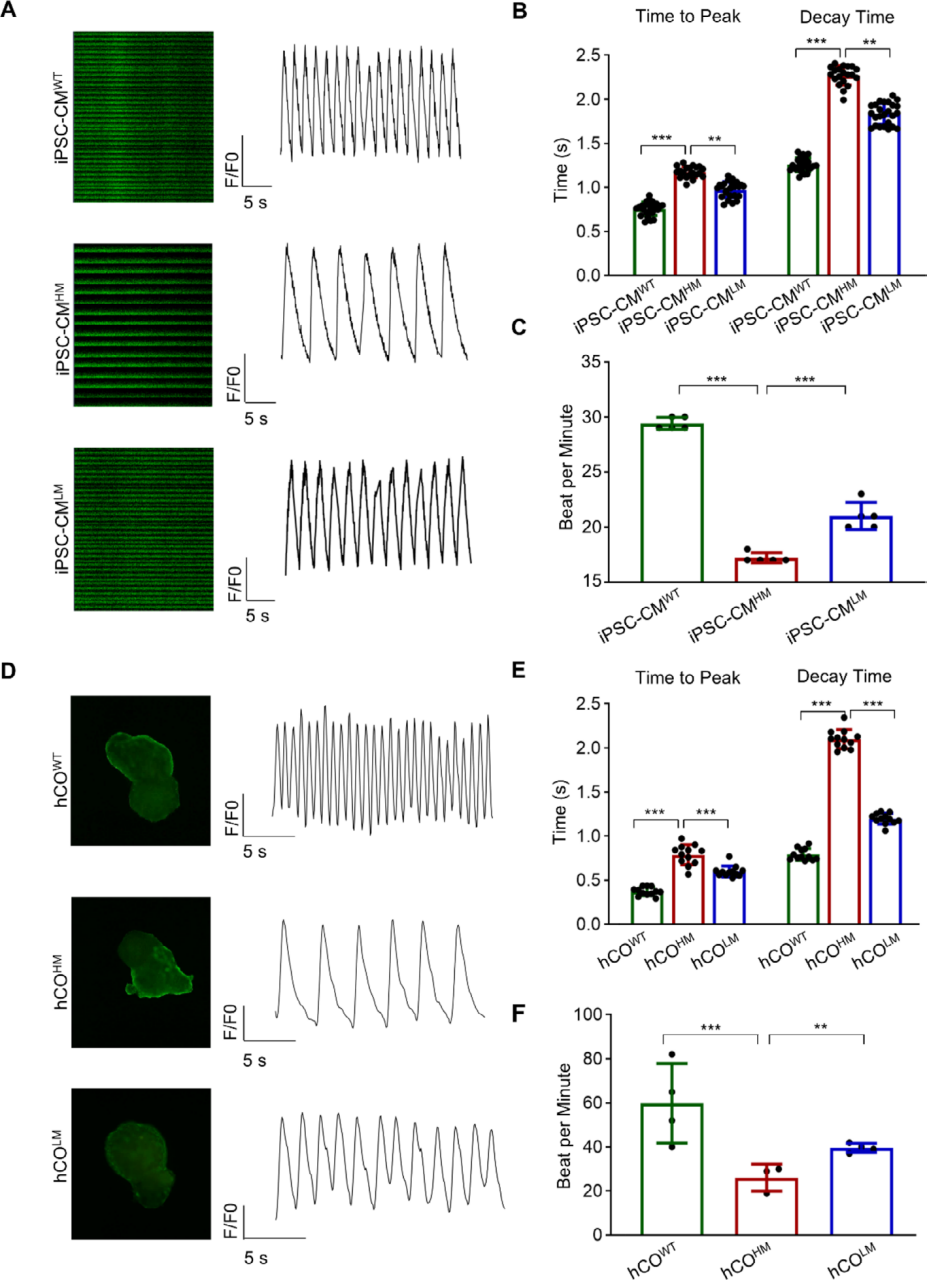
m.3243A>G mutation disrupted calcium homeostasis. (A) Calcium indicator Fluo‐4‐AM staining was used to indicate intercellular free calcium for cardiomyocytes. Analysis of calcium transient properties in iPSC‐CMs, including time to peak, decay time (B) and beat frequency (C). Similar Fluo‐4‐AM staining was conducted for cardiac organoids from iPSCs with different mutant frequencies, including time to peak, decay time (E) and beat frequency (F). Data are presented as mean ± SEM; one‐way ANOVA; **p < 0.01, ***p < 0.001.

Calcium handling is essential for cardiomyocyte function, with calcium transport being a key role of MAM. The disrupted MAM and the hypertrophic/senescent tendencies observed prompted an analysis of calcium signalling. We used the fluorescent calcium indicator Fluo‐4 AM to label intercellular free calcium. Compared to WT ones, cardiomyocytes with a high proportion of the m.3243A>G mutation exhibited prolonged calcium transient intervals (Figure 5A), characterised by longer time to peak and decay time (Figure 5B). Cardiac contractility, regulated by changes in intracellular calcium concentration, decreased beat frequency alongside prolonged calcium transients (Figure 5C), consistent with previous data (Movies [Link], [Link]). Mutation load reduction led to partial improvement in these indices (Figure 5A–C). Similar results were obtained from cardiac organoid experiments, where cardiac organoids from iPSC^HM^ displayed longer calcium transient intervals than those from iPSC^WT^ (Figure 5D–F). In conclusion, the m.3243A>G mutation disrupts calcium homeostasis, potentially leading to the disorganised sarcomere, hypertrophic phenotype and accelerated cellular ageing.

### 3.4 Enhancement of MAM Reversed the Phenotype Caused by m.3243A>G Mutation

**FIGURE 6:**
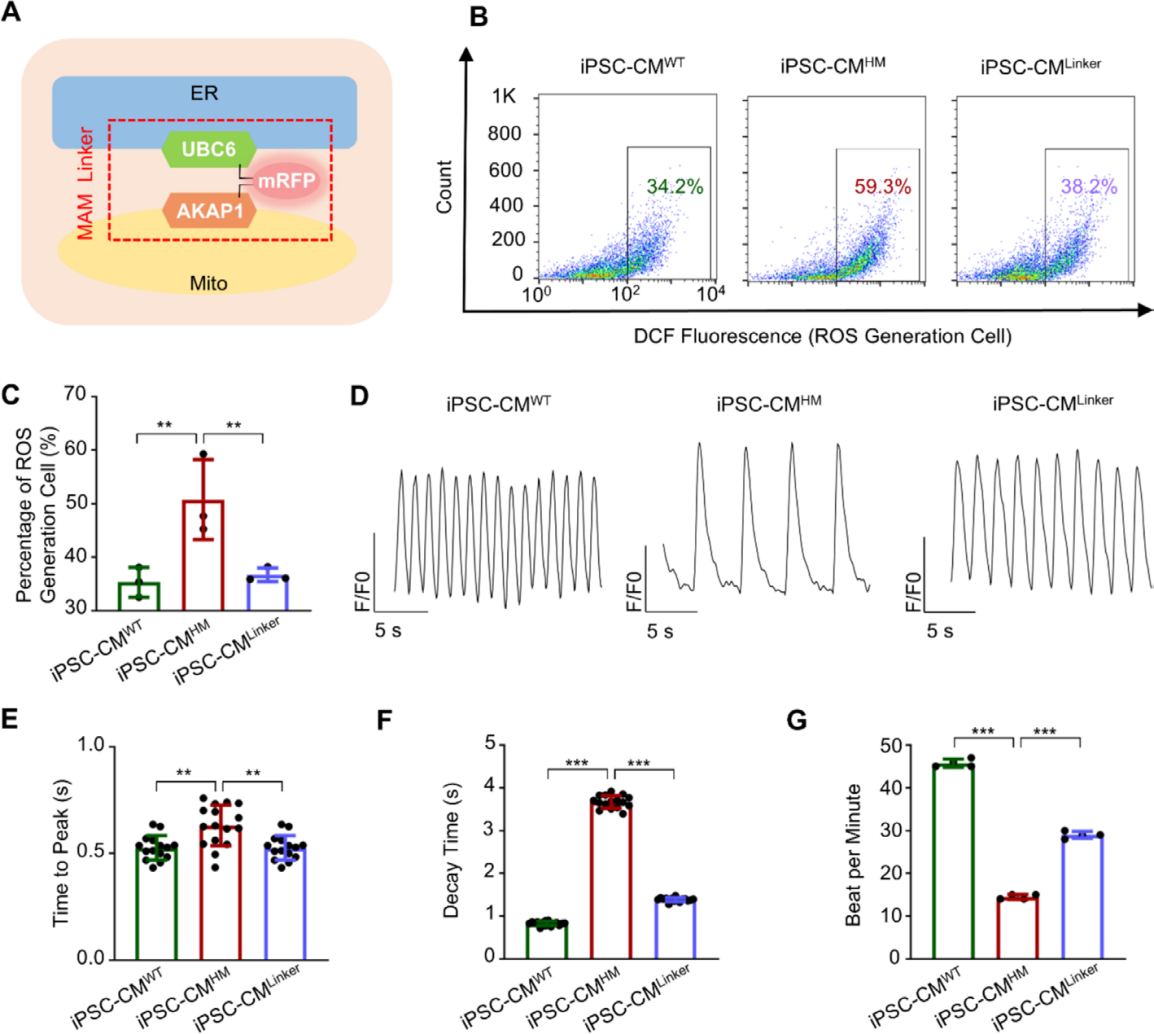
MAM Linker reversed the phenotype caused by the m.3243A>G mutation. (A) Schematic diagram of the MAM Linker. MAM linker contains three parts: The N‐terminal end of AKAP1 anchored to mitochondria (Mito), the C‐terminal end of UBC6 anchored to the endoplasmic reticulum (ER) and the middle segment mRFP. (B) Flow cytometry analysis was used to detect intercellular ROS accumulation in iPSC‐CM^WT^, iPSC‐CM^HM^ and iPSC‐CM^HM^ transfected with the MAM Linker. (C) Statistical analysis of intercellular ROS level. (D) Fluo‐4‐AM staining was used to detect calcium transients. Quantification of calcium transient properties, including time to peak (E), decay time (F) and beat frequency (G). Data are presented as mean ± SEM; one‐way ANOVA; **p < 0.01, ***p < 0.001.

To investigate whether the MAM disruption is directly linked to the phenotype caused by the m.3243A>G mutation, we reinforced the contact between ER and mitochondria by infecting cells with a MAM linker [47, 56, 57] (iPSC‐CM^Linker^). This synthetic plasmid is composed of three parts: the N‐terminal end encodes AKAP1, which localises to mitochondria; the C‐terminal end encodes UBC6, which localises to ER, and the middle segment encodes mRFP (Figure 6A). When expressed, this plasmid brings mitochondria and ER into closer proximity, thereby reinforcing MAM.

Enhancing MAM reversed several phenotypic abnormalities observed in iPSC‐CM^HM^, including reduced ROS production (Figure 6B,C), normalised calcium signalling, and restored beat frequency (Figure 6D–G). The cellular behaviours of iPSC‐CM^Linker^ closely resembled those of iPSC^WT^. These results suggest that MAM plays a crucial role in linking the m.3243A>G mutation to downstream cellular performance.

## 4 Discussion

Mitochondrial diseases are genetic disorders caused by mutations in mtDNA and/or nuclear DNA, resulting in dysfunction of the mitochondrial respiratory chain [58]. With advances in gene sequencing technologies and the investigation of large patient populations, the genetic background of mitochondrial disease has been rapidly elucidated. However, the pathological mechanisms underlying these diseases are still not well understood, limiting treatment strategies to symptomatic alleviation without curative options.

The m.3243A>G variant is the most common pathogenic mitochondrial mutation in clinical practice, associated with multi‐systemic manifestations including diabetes mellitus, hearing loss, visual impairment, encephalopathy, epilepsy, gastric and intestinal problems. Additionally, cardiac involvement is prevalent, with m.3243A>G carriers often suffering from cardiomyopathy [8, 59], left ventricular hypertrophy and rapid progression to heart failure [60, 61]. Investigating the involvement of the m.3243A>G mutation in cardiac issues has been challenging due to the lack of easily accessible disease models or samples. The shortage of animal models of mtDNA mutations arises mainly from the difficulty of engineering the mitochondrial genome [11, 62]. Successful efforts to directly model mtDNA mutations must overcome several major hurdles. Engineered mtDNA genomes must be able to traverse the mitochondrial membranes. To achieve a functional level of heteroplasmy, mutations must be targeted to a significant portion of the hundreds to thousands of copies of the mitochondrial genome in each cell. Although recombination between different mtDNA species occurs naturally, it is not yet feasible to harness this process as the mechanisms and regulation of mtDNA recombination are not well understood. Introduced mtDNA molecules that result in decreased respiratory capability often display a selective disadvantage and are selectively eliminated.

To our knowledge, no animal model has been reported where a specific, human‐disease‐based mtDNA mutation has been successfully recapitulated. The advent of the iPSC technique has revolutionised this field, enabling the modelling of cardiac diseases caused by genetic variations in vitro, which has been widely applied in mitochondrial disease research [63, 64].

In this study, we generated iPSCs carrying the m.3243A>G mutation and induced cardiac differentiation to investigate its effects on cardiomyocytes. Unlike the nuclear genome, mtDNA is polyploid, and pathogenic mutations often coexist with WT variants, a phenomenon known as mtDNA heteroplasmy [65]. We obtained iPSCs with varying proportions of mutation loads: a high mutation load (~80%) cell line (iPSC^HM^) and a mutation‐free cell line (iPSC^WT^) as isogenic control. We also generated an iPSC cell line with a very high mutation load (> 90%), but it could not be differentiated into cardiomyocyte, which echoes the previous finding that a high mutation load inhibits cardiac development [66].

To reduce the heteroplasmy level in iPSC^HM^, we have engineered mitochondrially targeted transcription activator‐like effector nucleases (mito‐TALENs) to selectively eliminate mutant mtDNA, resulting in another cell line (iPSC^LM^) with a reduced heteroplasmy (~30%) [49]. Both iPSC^HM^ and iPSC^LM^ were derived from the same origin but differed in mutation burden while maintaining the same nuclear DNA background. The mutation rate remained relatively stable without significant heteroplasmic shifts during subsequent cultivation and differentiation, consistent with previous studies [67, 68]. Moreover, varying heteroplasmy levels did not affect the efficiency of cardiac differentiation, as mutation loads under 90% do not impair iPSC differentiation into cardiomyocytes [66]. This enabled us to compare cellular phenotypes across varying mutation loads and uncover underlying mechanisms. The iPSC‐CM^HM^ exhibited a hypertrophic phenotype and accelerated ageing. Conversely, when the heteroplasmy level was reduced below the threshold, iPSC‐CM^LM^ cellular behaviour resembled that of iPSC‐CM^WT^. It should be noted that more clinical samples are required to excluderule out patient or cell specificity, which is a focus of our future work.

We initially investigated the structure of tRNA^Leu(UUR)^ due to the m.3243A>G mutation localised to the D‐loop of tRNA^Leu(UUR)^. Molecular dynamic simulations revealed that the mutation loosened and destabilised tRNA^Leu (UUR)^, leading to mitochondria dysfunction, as observed with other mtDNA mutations [69, 70]. Previous studies on *trans* mitochondrial cybrid cells suggested deficient aminoacylation of mutant mt‐tRNA^Leu (UUR)^ and defective mitochondrial protein synthesis [71, 72, 73]. The iPSC‐CM^HM^ exhibited severe respiratory chain deficiency, particularly in Complexes I, II, and IV, replicating clinical manifestation observed in the brains of m.3243A>G carriers diagnosed with MELAS [74]. Consequently, iPSC‐CM^HM^ showed defective oxidation phosphorylation activity, reduced ATP production, diminished mitochondrial membrane potential and increased ROS accumulation, indicating overall mitochondrial dysfunction, which has been confirmed in iPSC‐derived endothelial cells [75], retinal pigment epithelial cells [76] and neurons [77]. Mitochondria in cardiomyocytes bearing the m.3243A>G mutation became rounded, similar to those in lymphoblasts bearing mtDNA mutation [69]. Recently, another group reported severe cardiomyopathy in a patient with high m.3243A>G load, with patient‐specific iPSC‐CMs showing similar phenotypes to those in this article [78]. However, this work merely described cellular‐level symptoms without revealing mechanisms.

Beyond mitochondria, we investigated its interaction with ER, specifically the MAMs. MAM is a physical connection that is beneficial for efficient calcium trafficking from the ER to mitochondria [24]. Tracking mitochondria and ER interactions revealed weakened interaction between them, confirmed by statistical analysis following scanning electron microscopy and machine learning. This was manifested by decreased contact length and contact number. The reduced contact incidence may result from changes in mitochondrial shape, reducing the absolute number or relative contact area. Studies indicate that contact sites between ER sheets and mitochondria regulate mtDNA replication and segregation [79], highlighting an interactive and reciprocal relationship. Constitutive activation of the PI3K‐Akt‐mTORC1 pathway was predicated on sustaining the m.3243 A>G mtDNA mutation by disturbing mitochondrial quality control [80]. Consistent with disrupted MAMs, ER–mitochondria calcium trafficking was blocked, leaving calcium detained in ER, potentially causing an imbalance in calcium homeostasis in mitochondria and cytosol. The overexpressed MAM Linker in iPSC‐CM^HM^ reinforced ER–mitochondria interaction, partially restoring cellular phenotypes to control ones. In this work, it is the MAM we focused on. We cannot deny other factors that can affect calcium transient, including L‐type calcium channels, the Na^+^/Ca^2+^ exchanger, phosphatases and protein kinases, hormones and neurotransmitters, intracellular signalling pathways and extracellular environmental factors [81]. To our knowledge, this is the first application of machine learning for MAM analysis in the context of mitochondrial mutant‐associated cardiovascular disease. Unfortunately, we could not determine changes in the proteome localised in MAM due to the difficulty of isolating MAM from iPSC‐CMs. Future studies will focus on how mtDNA affects nuclear DNA to elucidate the molecular mechanism of the m.3243 A>G mutation and reveal the pathogenic mechanism of MIDD.

**FIGURE 7:**
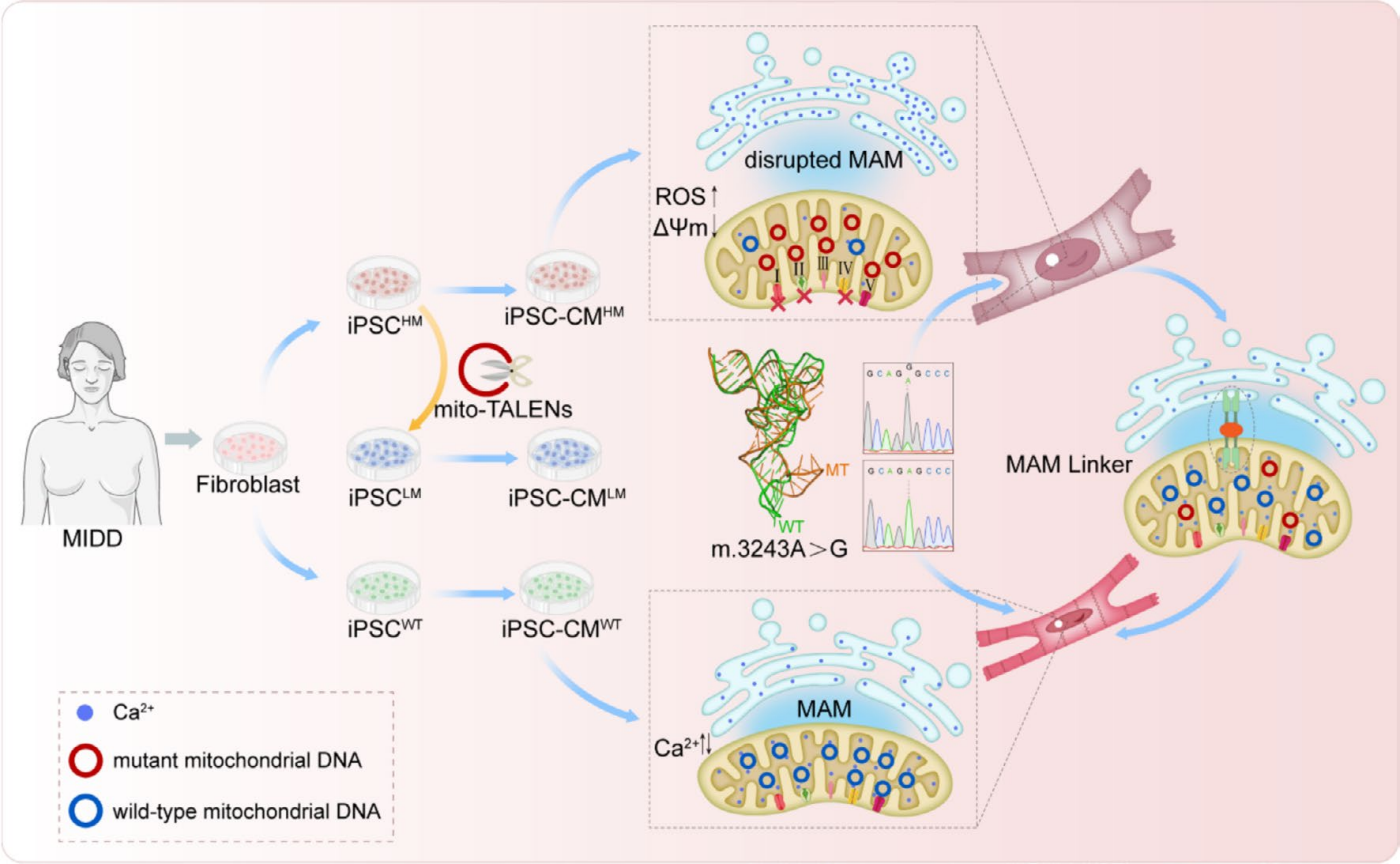
Working model. In this study, maternally inherited diabetes and deafness (MIDD) patient‐specific induced pluripotent stem cells (iPSCs) with a high proportion of m.3243A>G mutation (iPSC^HM^) or without mutation (iPSC^WT^) were established by fibroblast reprogramming. The mito‐TALENs were adopted to digest mutant mitochondria in iPSC^HM^, and an iPSC cell line with a relatively low mutation load (iPSC^LW^) was then generated. The three abovementioned cell lines were induced directly toward cardiomyocytes, named iPSC‐CM^HM^, iPSC‐CM^WT^ and iPSC‐CM^LW^, respectively. Cardiomyocytes with a high m.3243A>G mutation load exhibited hypertrophic phenotype. Adenine at position 3243 of mtDNA localised in the gene *MT‐TL1* encoding tRNA^Leu (UUR)^. The m.3243A>G mutation altered the conformation of tRNA^Leu (UUR)^. Mitochondria in cardiomyocytes with a high proportion of m.3243A>G mutation dysfuntioned, manifested by defective oxidation phosphorylation, leading to ATP underproduction, ROS accumulation, and membrane potential reduction. As a dynamic organelle, its interaction with ER, namely mitochondria‐associated ER membrane (MAM), was disrupted consequently. The disrupted MAM disturbed calcium homeostasis, which may be the direct and leading cause of cardiomyocyte hypertrophy, as MAM enforcement reversed this pathological state.

In conclusion, this study demonstrated that cardiomyocytes carrying the m.3243A>G mutation exhibit a hypertrophic phenotype due to disrupted MAM formation. The mutation alters the conformation of tRNA^Leu (UUR)^, destabilising it and leading to mitochondrial dysfunction. With impaired mitochondrial function, the mitochondrial architecture is changed, and their physical interaction with ER (MAM) is weakened. As MAM serves as a hub for calcium signalling, disrupted MAM interferes with calcium trafficking, resulting in hypertrophic cardiomyocytes with disorganised sarcomeres. After reducing the mutation load with mito‐TALENs, the ER–mitochondria interaction improved, and cellular behaviour tended to be normal. In conclusion, maintaining proper interaction between mitochondria and ER may provide a novel perspective for intervention in mitochondrial diseases (Figure 7).

## Author Contributions

S.H., W.L. and M.Y. conceived and designed the project. S.H., W.L. and M.Y. supervised the experiments. J.Z. was invovled in the disscussion of project. S.H., Z.S., W.L. and M.Y. wrote and revised the manuscript. M.Y. and M.S. acquired and analysed the majority of the data. M.S. and S.C. conducted cellular experiments. M.Z. collected clinical sample and performed clinical phenotype assessments. Y.F. provided plasmids for mito‐TALENs. B.N. and X.L. performed biological and molecular experiments.

## Conflicts of Interest

The authors declare no conflicts of interest.

## Supporting information


**Data S1.** Supporting Information.

**Movie S1.** Video record of beating iPSC‐CM^WT^.

**Movie S2.** Video record of beating iPSC‐CM^HM^.

**Movie S3.** Video record of beating iPSC‐CM^LM^.

## Data Availability

The data that support the findings of this study are available on request from the corresponding author. The data are not publicly available due to privacy or ethical restrictions.
