# Supplementary material for: Mitochondrial Mutation Leads to Cardiomyocyte Hypertrophy by Disruption of Mitochondria‐Associated ER Membrane
Source: Cell Prolif. 2025 Feb 21;58(7):e70002. doi: 10.1111/cpr.70002 (PMC12240637; doi:10.1111/cpr.70002)
Supplement: Supplementary file 1 — Data S1. Supporting Information. [file CPR-58-e70002-s003.docx]

**Supplementary Information**

**Mitochondrial mutation leads to cardiomyocyte hypertrophy by disruption of mitochondria-associated ER membrane**

Miao Yu^1, #^, Min Song^1, #^, Manna Zhang^2, #^, Baoqiang Ni^1^, Shuangshuang Chen^1^, Xuechun Li^1^, Wei Lei^1^, Zhenya Shen^1^, Yong Fan^3^, Jianyi Zhang^4, 5^, Shijun Hu^1, *^

^1^ Department of Cardiovascular Surgery of the First Affiliated Hospital & Institute for Cardiovascular Science, Collaborative Innovation Center of Hematology, State Key Laboratory of Radiation Medicine and Protection, Suzhou Medical College, Soochow University, Suzhou, Jiangsu 215000, China; ^2^ Department of Endocrinology and Metabolism, Shanghai Tenth People’s Hospital, School of Medicine, Tongji University, Shanghai, Shanghai 200072, China; ^3^ Department of Obstetrics and Gynecology, Guangdong Provincial Key Laboratory of Major Obstetric Diseases, Guangdong Provincial Clinical Research Center for Obstetrics and Gynecology, Guangdong-Hong Kong-Macao Greater Bay Area Higher Education Joint Laboratory of Maternal-Fetal Medicine, the Third Affiliated Hospital of Guangzhou Medical University, Guangzhou, Guangdong 510150, China; ^4^ Department of Biomedical Engineering, School of Medicine and School of Engineering, The University of Alabama at Birmingham, Birmingham, AL 35233, United States; ^5^ Department of Medicine, Division of Cardiovascular Disease, School of Medicine, The University of Alabama at Birmingham, Birmingham, AL 35233, United States.

**^#^ Co-first authors**

**Supplemental Figures & Figure Legends**

| **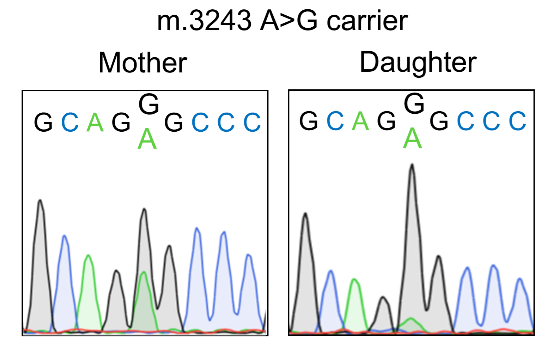** |
| --- |
| **Supplemental Figure S1. Sanger sequencing of the skin fibroblast of the sampled MIDD patient**. |

| 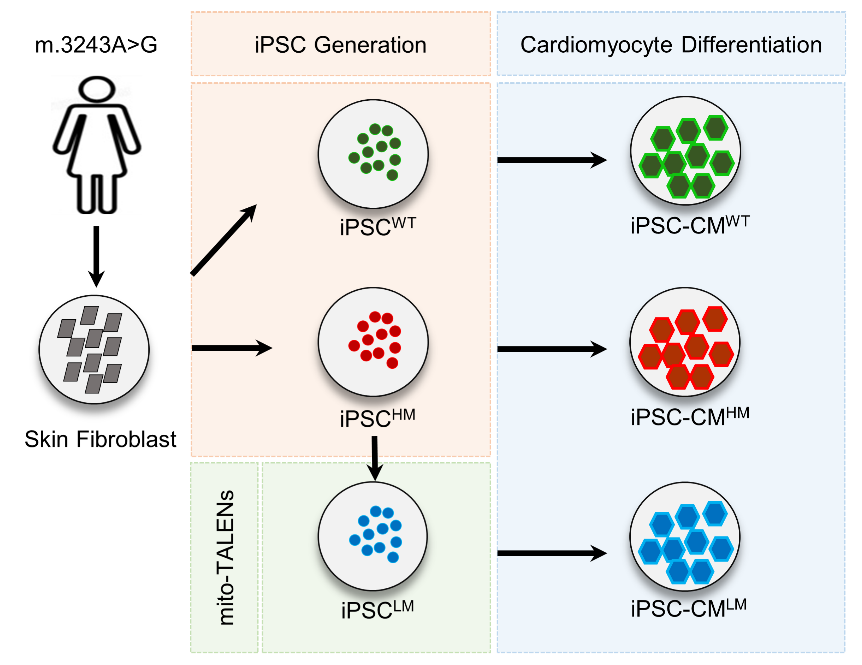 |
| --- |
| **Supplemental Figure S2. Schematic workflow of iPSC generation and cardiomyocyte differentiation.** MIDD patient-specific induced pluripotent stem cells (iPSCs) with a high proportion of m.3243A>G mutation (iPSC^HM^) or without mutation (iPSC^WT^) were established by fibroblast reprogramming. mito-TALENs was adopted to digest mutant mitochondria in iPSC^HM^, and iPSC cell line with a relatively low mutation load (iPSC^LW^) was then generated. The three abovementioned cell lines were differentiated into cardiomyocytes, named iPSC-CM^HM^, iPSC-CM^WT^, and iPSC-CM^LW^_,_ respectively. |

| 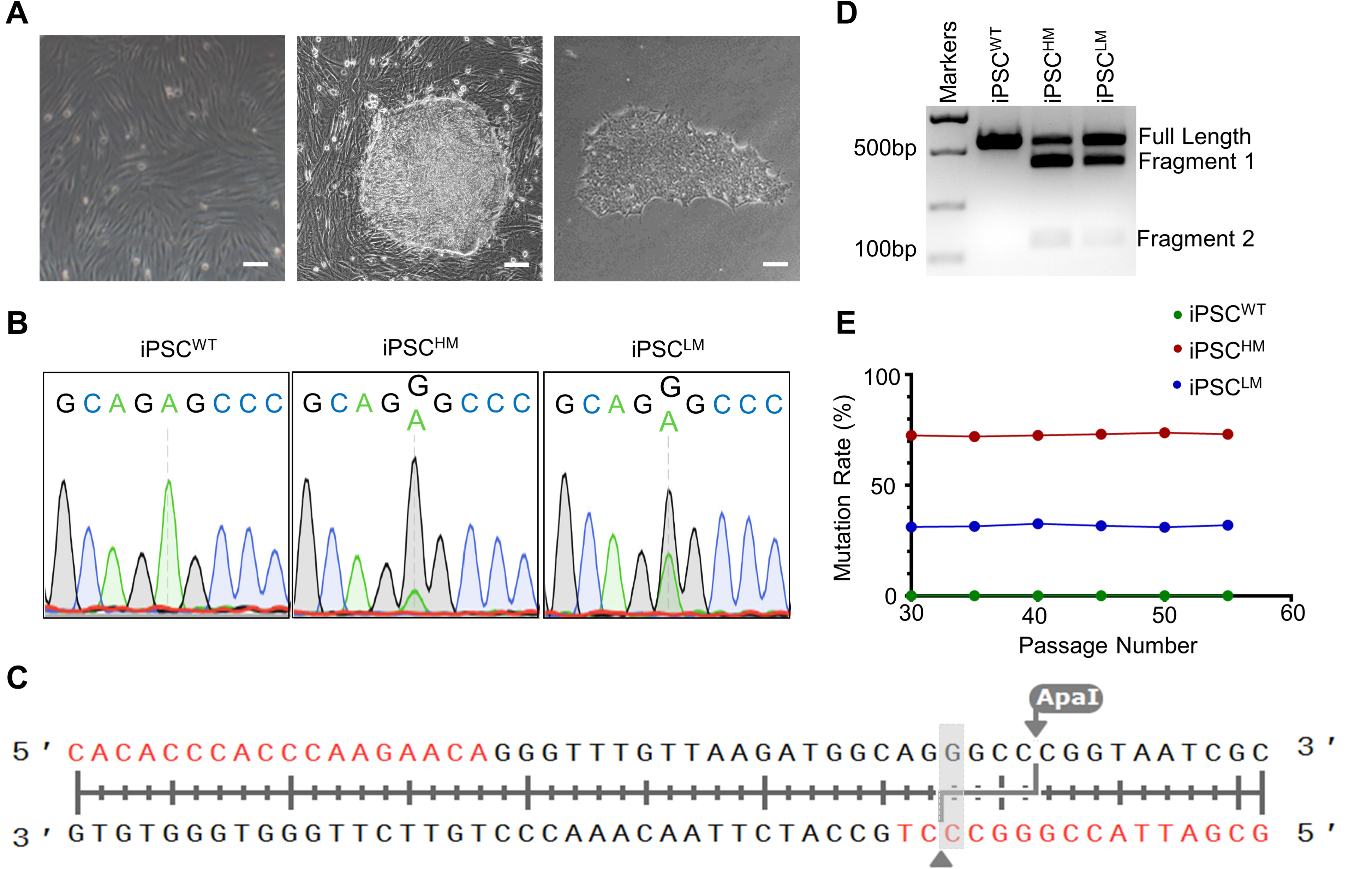 |
| --- |
| **Supplemental Figure S3. iPSC reprogramming and mutation rate confirmation.** (A) Fibroblast reprogramming to generate iPSC clones. Scale bar: 100 μm. (B) Sanger sequence results suggested m.3243A>G mutation of *MT-TL1* gene in iPSC^HM^ and iPSC^LM^. (C) The mtDNA sequences are shown, with sequences recognized and bound by mito-TALENs in red and the ApaI cleavage site in darker parts. (D) PCR-RFLP was used to detect the m.3243A>G point mutation rate in iPSCs. (E) Statistics of m.3243A>G mutation rate in different passages of iPSCs. |

| 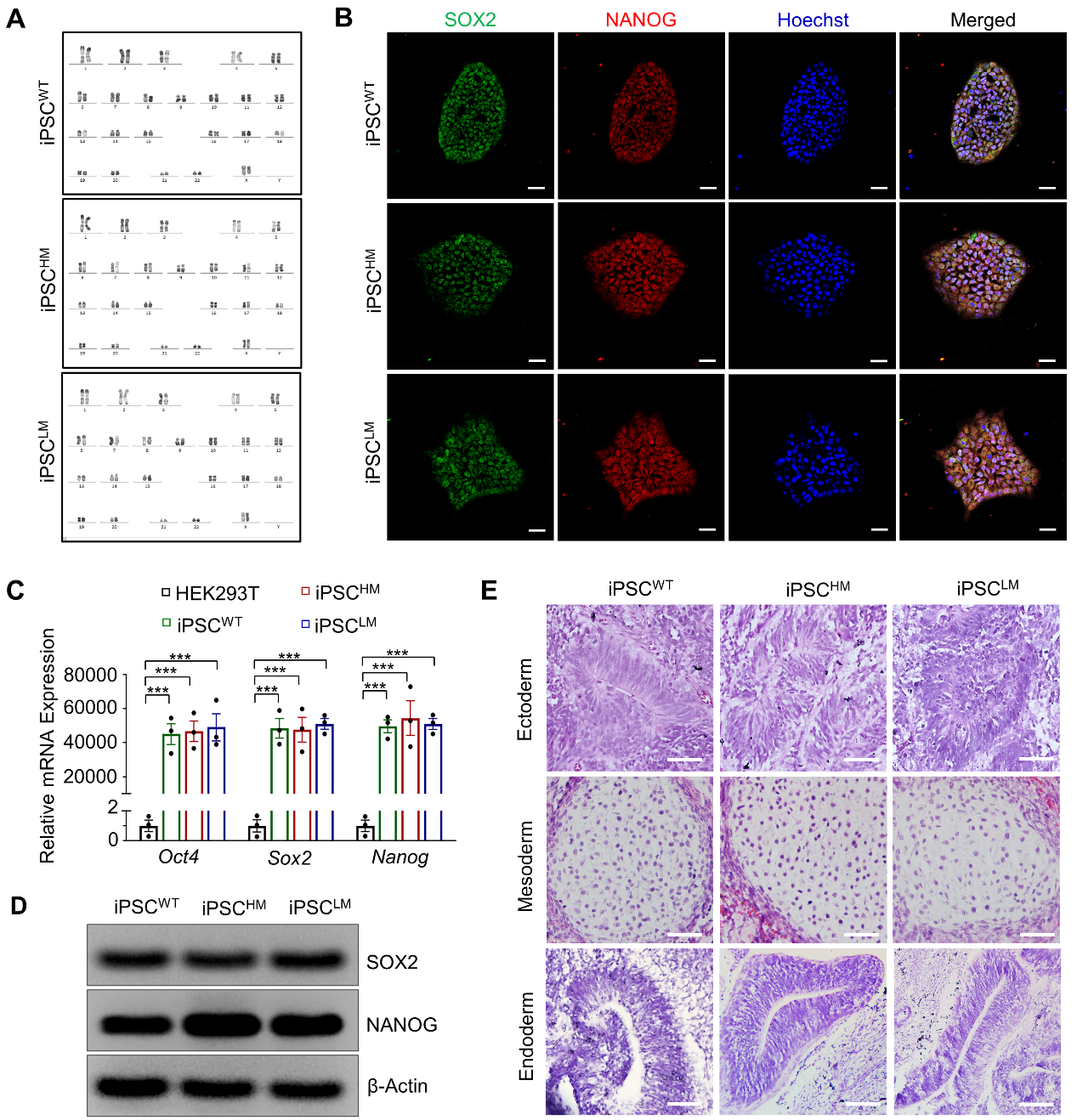 |
| --- |
| **Supplemental Figure S4. Characterization of patient-specific iPSCs.** (A) Karyotype analysis was used to detect chromosome number and structure. (B) The iPSC^WT^, iPSC^HM,^ and iPSC^LM^ were immunostained with pluripotent markers (SOX2 & NANOG), and Hoechst 33342 was used for nuclear probing. Scale bar: 50 μm. (C) Real-time PCR was used to detect the mRNA levels of stemness markers *Oct4*, *Sox2*, and *Nanog*. (D) Western Blot was used to detect the protein levels of stemness markers SOX2 and NANOG. (E) HE staining of teratomas indicated that iPSC^WT^, iPSC^HM^, and iPSC^LM^ were able to differentiate into ectoderm, mesoderm, and endoderm. Scale bar: 50 μm. Data are presented as mean ± SEM; one-way ANOVA; ***p<0.001. |

| 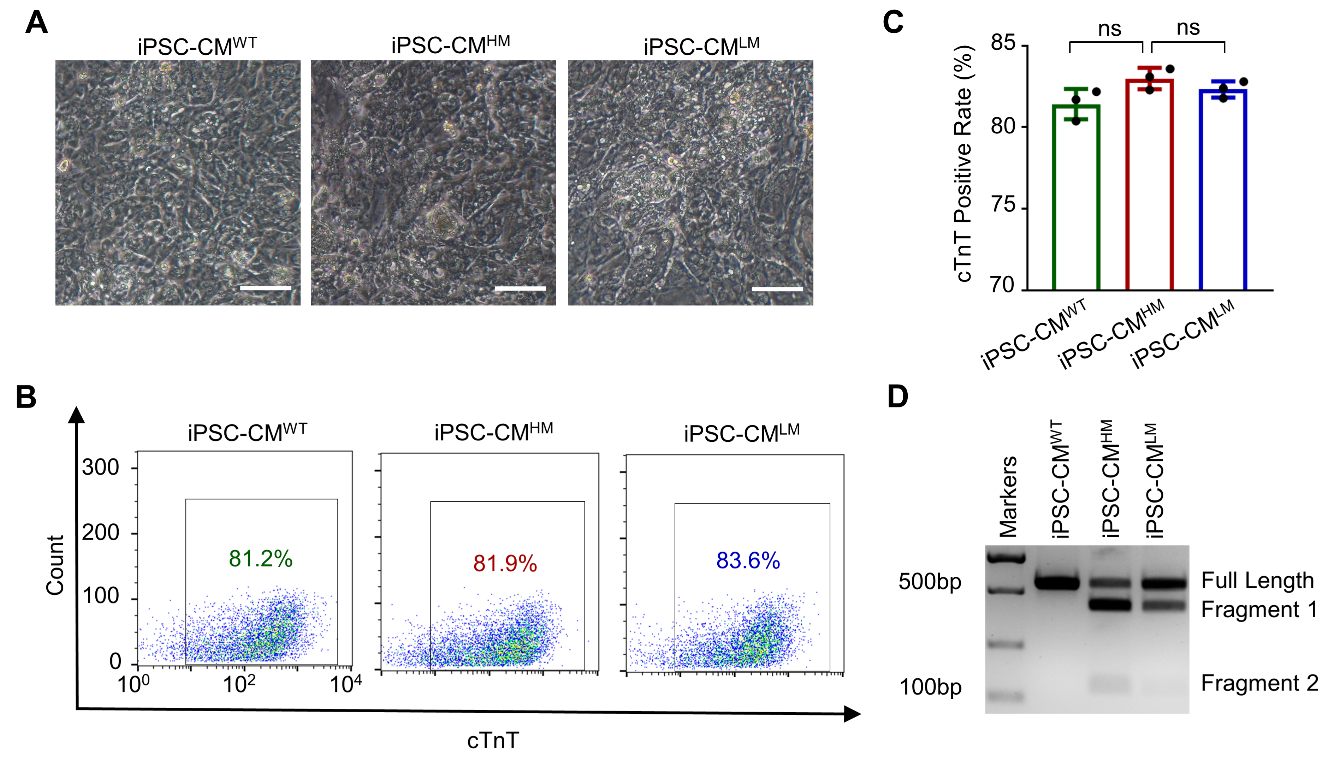 |
| --- |
| **Supplemental Figure S5. iPSC^m.3243A>G^ derived cardiomyocytes.** (A) Images of cardiomyocytes obtained by induced differentiation of iPSC^WT^, iPSC^HM^, and iPSC^LM^. Scale bar:100 μm. (B) Flow cytometry analysis was used to detect the differentiation efficiency of iPSC-derived cardiomyocytes. (C) Statistical analysis of panel B. (D) PCR-RFLP was used to detect the m.3243A>G point mutation rate in cardiomyocytes. Data are presented as mean ± SEM; one-way ANOVA; ns: not significant. |

| 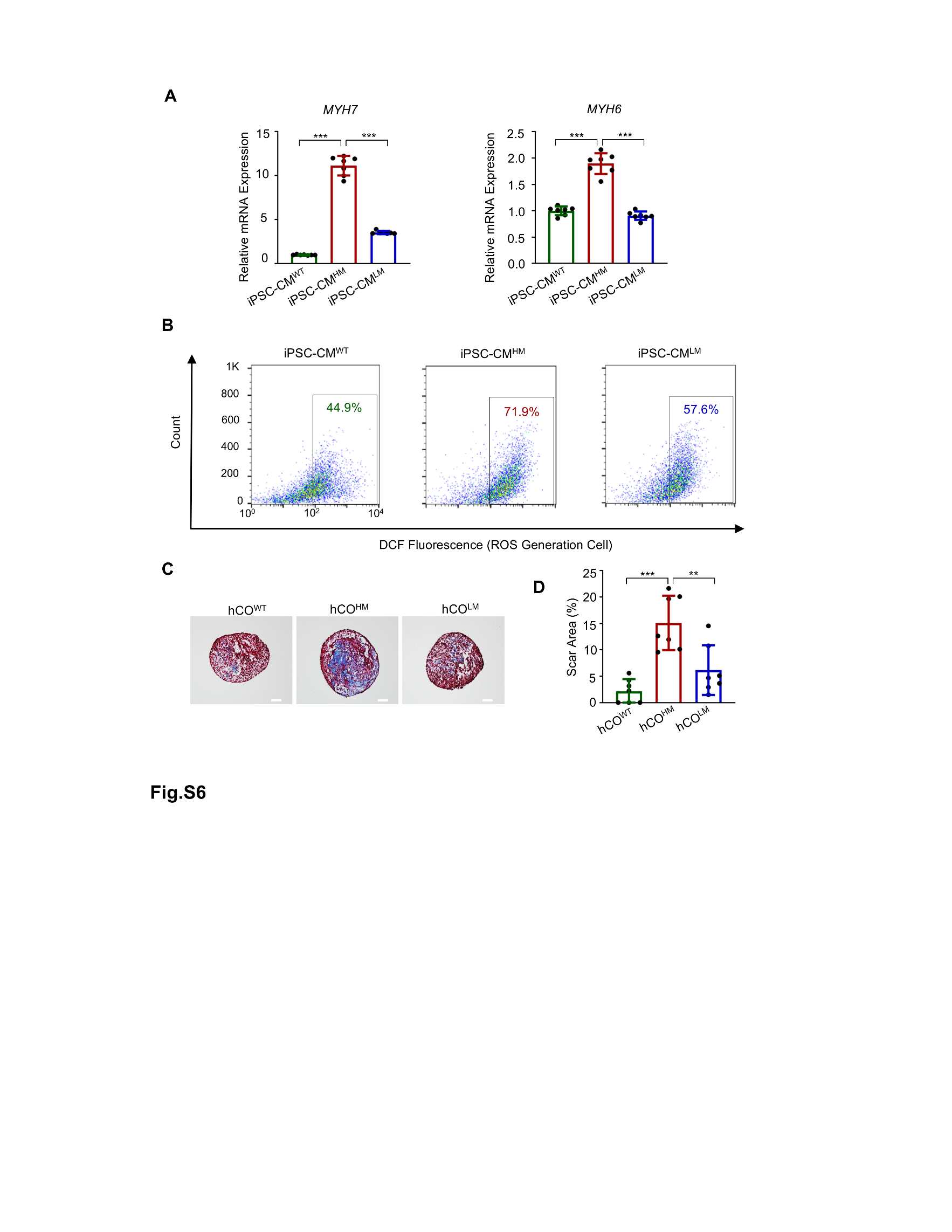 |
| --- |
| **Supplemental Figure S6. m.3243 A>G mutation induced cardiomyocyte hypertrophy.** (A) Real-time PCR was used to detect the expression of *MYH7* and *MYH6* normalized to *18S rRNA*. (B) DCFH-based flow cytometry analysis was used to detect cardiomyocyte ROS levels. (C) Masson staining of human cardiac organoids (hCOs) was used to detect fibrosis. Scale bar: 50 μm. (D) Statistical analysis of scar area in panel C. Data are presented as mean ± SEM; one-way ANOVA; **p<0.01, ***p<0.001. |

| **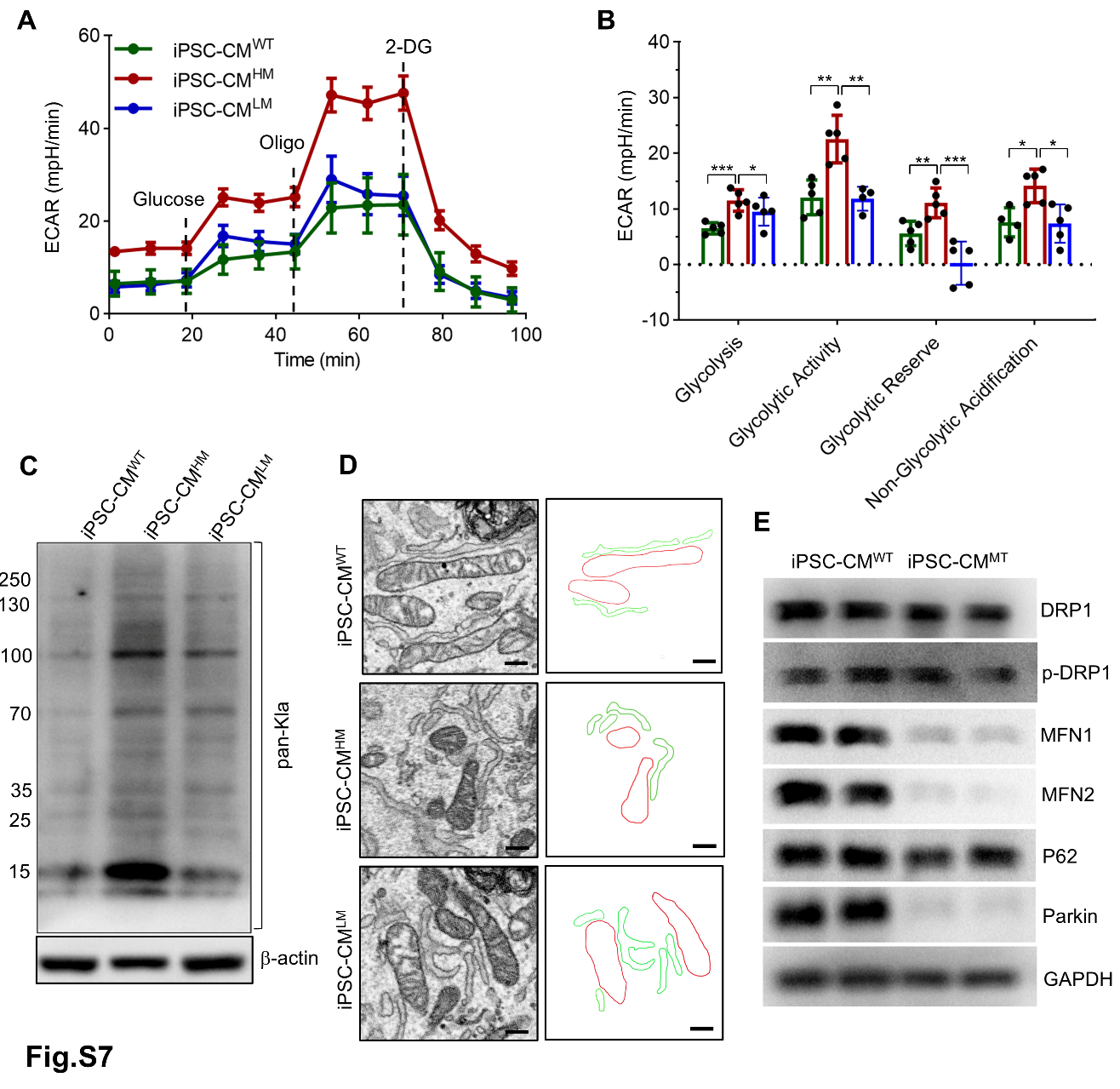** |
| --- |
| **Supplemental Figure S7. m.3243 A>G mutation induced enhanced glycolysis.** (A) Seahorse XF assay was used to evaluate EACR for cellular glycolysis. (B) Statistical analysis of glycolysis, glycolytic activity, glycolytic reserve, and non-glycolytic acidification. (C) Western Blot was used to detect lysine lactylation. (D) Schematic diagram of mitochondria and endoplasmic reticulum contact. Scale bar: 1 μm. (E) Western Blot was used to detect mitochondria dynamics and mitophagy. Data are presented as mean ± SEM; one-way ANOVA; *p<0.05, **p<0.01, ***p<0.001. |

| **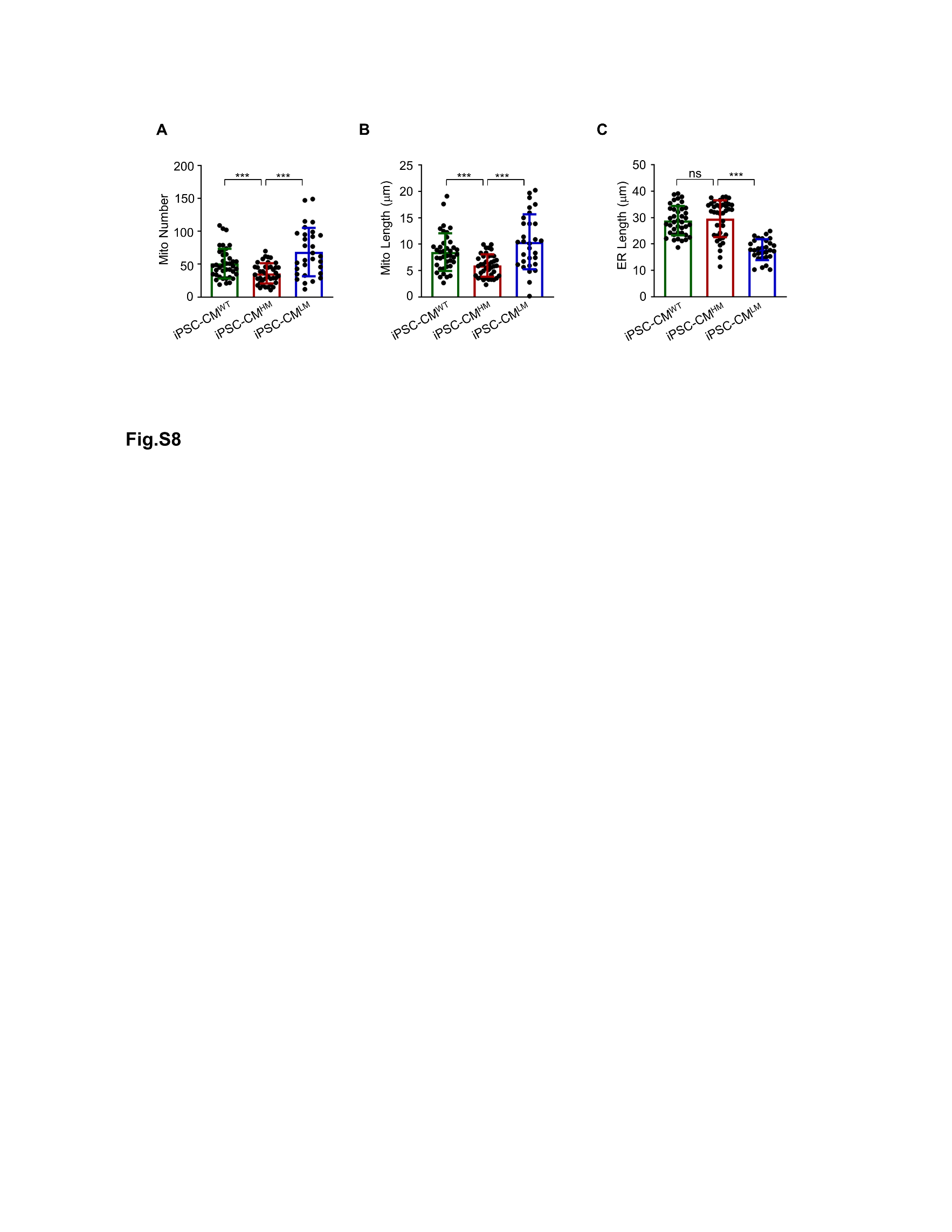** |
| --- |
| **Supplemental Figure S8. m.3243 A>G mutation disrupted the association between mitochondria and ER.** (A) Statistical analysis of Mito number in iPSC-CM^WT^, iPSC-CM^HM^, and iPSC-CM^LM^. (B) Statistical analysis of Mito length. (C) Statistical analysis of ER length. Data are presented as mean ± SEM; one-way ANOVA; ***p<0.001, ns: not significant. |

**Supplemental Table 1. Antibodies used in the study.**

| **Antibody name** | **Company, Catalog Number.** | **Dilution** |
| --- | --- | --- |
| SOX2 | Santa Cruz, sc-365823 | 1:300 for IF; 1:3000 for WB |
| NANOG | Santa Cruz, sc-33759 | 1:300 for IF; 1:3000 for WB |
| Troponin T (TNNT2) | Proteintech,15513-1-Ap | 1:300 for IF; 1:200 for FCM; |
| Alex Fluor 594_Donkey Anti-Mouse IgG(H+L) | Jackson ImmunoResearch, 715-585-150 | 1:500 for IF |
| Alex Fluor 488_Donkey Anti-Rabbit IgG(H+L) | Jackson ImmunoResearch, 711-545-152 | 1:500 for IF |
| Goat anti-Mouse IgG (H+L)-HRP | Jackson ImmunoResearch, 115-035-003 | 1:3000 for WB |
| Goat Anti-Rabbit IgG (H+L)-HRP | Jackson ImmunoResearch, 111-005-045 | 1:3000 for WB |
| α-actinin | Sigma-Aldrich, A7811 | 1:300 for IF |
| β-Actin | Affinity, T0022 | 1:3000 for WB |
| Oct3/4 | Santa Cruz, sc-5279 | 1:3000 for WB |
| Total OXPHOS Human WB Antibody Cocktail | Abcam, ab110411 | 1:2000 for WB |
| L-Lactyl Lysine | PTM BIO, PTM-1401RM | 1:2000 for WB |
| Immunofluorescence (IF); Western blot (WB); Flow cytometry (FCM). | | |

**Supplemental Table 2. Primers used for real-time PCR in the study.**

| **Targets** | **Primer sequence (5'-3')** | **Product Length (bp)** |
| --- | --- | --- |
| *SOX2* | F-GCCGAGTGGAAACTTTTGTCG | 154 |
|  | R-GCAGCGTGTACTTATCCTTCTT |  |
| *OCT4* | F-GTGGAGGAAGCTGACAACAA | 120 |
|  | R-ATTCTCCAGGTTGCCTCTCA |  |
| *NANOG* | F-ATAGATAAGTAGATCTAATAC | 195 |
|  | R-AAACGGTAAGAAATCAATTAA |  |
| *P16* | F-CTTCGGCTGACTGGCTGG | 129 |
|  | R-TCATCATGACCTGGATCGGC |  |
| *P21* | F-CACCTCACCTGCTCTGCTGC | 151 |
|  | R-GCTGGTCTGCCGCCGTTTT |  |
| *MYH6* | F-CGGCCCTTTGACATTCGCACT | 131 |
|  | R-TTCACAGTCACCGTCTTCCCA |  |
| *MYH7* | F-AACGACAACTCCTCCCGCTTC | 188 |
|  | R-ATGTCCAGCAGCTCAGGCTTT |  |
| F, forward; R, reverse. | | |

**Supplemental Movie 1.** Video record of beating iPSC-CM^WT^.

**Supplemental Movie 2.** Video record of beating iPSC-CM^HM^.

**Supplemental Movie 3.** Video record of beating iPSC-CM^LM^.
